# Positive Association of Cardiovascular Disease (CVD) with Chronic Exposure to Drinking Water Arsenic (As) at Concentrations below the WHO Provisional Guideline Value: A Systematic Review and Meta-analysis

**DOI:** 10.3390/ijerph17072536

**Published:** 2020-04-07

**Authors:** Lingqian Xu, Debapriya Mondal, David A. Polya

**Affiliations:** 1 Department of Earth and Environmental Sciences and Williamson Research Centre for Molecular Environmental Science, University of Manchester, Manchester M13 9PL, UK; lingqian.xu@postgrad.manchester.ac.uk (L.X.); david.polya@manchester.ac.uk (D.A.P.); 2 School of Science, Engineering and Environment, University of Salford, Salford M5 4WT, UK

**Keywords:** arsenic, low level, cardiovascular disease, dose-response, systematic review, meta-analysis

## Abstract

To the best of our knowledge, a dose-response meta-analysis of the relationship between cardiovascular disease (CVD) and arsenic (As) exposure at drinking water As concentrations lower than the WHO provisional guideline value (10 µg/L) has not been published yet. We conducted a systematic review and meta-analyses to estimate the pooled association between the relative risk of each CVD endpoint and low-level As concentration in drinking water both linearly and non-linearly using a random effects dose-response model. In this study, a significant positive association was found between the risks of most CVD outcomes and drinking water As concentration for both linear and non-linear models (*p*-value for trend < 0.05). Using the preferred linear model, we found significant increased risks of coronary heart disease (CHD) mortality and CVD mortality as well as combined fatal and non-fatal CHD, CVD, carotid atherosclerosis disease and hypertension in those exposed to drinking water with an As concentration of 10 µg/L compared to the referent (drinking water As concentration of 1 µg/L) population. Notwithstanding limitations included, the observed significant increased risks of CVD endpoints arising from As concentrations in drinking water between 1 µg/L and the 10 µg/L suggests further lowering of this guideline value should be considered.

## 1. Introduction

Originating from either geological or anthropogenic activities, arsenic (As) has been widely recognized since the 1950s as one of the most serious human carcinogens [1,2]. Ubiquitously present in the environment [3,4,5], exposure to As can take place via ingestion (oral), dermal contact, inhalation, and even parenteral routes [6], and can lead to a wide range of carcinogenic and non-carcinogenic end-points [7,8]. Long-term As exposure is known to result in lung, bladder, liver, skin and kidney cancers [9]. Concerning non-carcinogenic endpoints, while skin lesions are considered to be a primary marker of As toxicity [10], it has also been reported to be associated with different neurological problems [11], increased risk of immune problems in new-born [12], infant infections [13], some reproductive health problems in pregnant women [14] and, importantly, cardiovascular diseases (CVD). A prospective study conducted in Bangladesh found an increased risk of mortality from ischemic heart disease and cerebrovascular disease in populations exposed to high concentration of inorganic arsenic (iAs) in well water [15]. Similarly, in other studies, increased risk of hypertension [16], stroke [17] and changes in CVD biomarker levels [6,18] were observed associated with As exposure. Among all the adverse health risks from As exposure, CVD is regarded as the most serious non- carcinogenic detrimental health outcome [15,19].

The relationship between CVD and As exposure at As concentrations in drinking water greater than 100 µg/L is well established [17,18,20,21]. However, partly as a result of the joint effort by Environmental Protection Agency and National Academy of Sciences to define an As risk threshold, the need to determine the dose-response at lower concentrations is also well recognized [22]. Unfortunately, evidence for low concentration effects, especially below the WHO permissible limit (10 µg/L) has been controversial and inconsistent [23,24]. For example, a cross-sectional study conducted by Kunrath et al. [25] found an association between CVD risks and low-level As exposure, while other studies such as that of Jones, et al. [26] for hypertension and that of Monrad et al. [27] for myocardial infarction did not find similar associations. Such inconsistency might be related to limitations with sample size, exposure and outcome assessment as well as insufficient information on different CVD risk factors [26,28,29]. As the available scientific data do not allow characterization of a clear threshold below which CVD risk would be negligible [23,29] and the disagreements between individual studies are a clear problem for regulators who currently face mounting pressure to determine “better” regulatory limits for As in drinking water, the National Research Council has recommended the use of meta-analysis to quantify the dose-response relationship of different CVD endpoints to low-level As exposure [30]. Compared to individual studies where detecting the possible true effects is often prevented due to their relatively small number of participants, meta-analysis could lower the background noise, reduce error and bias and is a rigorous, transparent and systematic approach in evaluating aggregated evidence to answer a specific question [31,32].

Existing meta-analyses and systematic reviews that include the lower end of the As exposure range have not clearly and quantitatively specified any increased risk below 10 µg/L of water As concentration possibly because of a lack of epidemiological data. These reviews notably include a) a qualitative systematic review of the epidemiologic evidences on the association of CVD and low, moderate and high As exposure [33], b) a meta-regression of the association between risk of CVD and As exposure at concentrations lower than those recommended by previous studies [34], c) shaping the dose-response effects on incidence of CVD endpoints of chronic exposure to low-moderate (< 100 µg/L) and high levels (≥ 100 µg/L) of As [24], d) unveiling the epidemiologic evidence on the relationship between CVD and As exposure in studies that include the lower end of the exposure range [22], and e) qualitatively identifying an association between the prevalence of hypertension and both low and moderate-to-high As exposure [35].

Due to the recent publication of a number of key epidemiological studies [36] building upon our recent research in exposure science [37,38,39], we aimed to use meta-analysis to determine quantitatively the magnitude of increased CVD risks for the general population exposed at As concentrations lower than the WHO provisional guideline value for drinking water of 10 µg/L. We have also included some CVD biomarkers in the evaluation.

## 2. Materials and Methods

### 2.1. Data Sources

We searched ISI Web of Science for studies published before December 2019 on the association between CVD risks including overall CVD, coronary heart disease (CHD), stroke, carotid atherosclerosis disease, hypertension and associated clinic markers and As exposure and for the general population. The medical subject headings (MeSH) used were “arteriosclerosis”, “atherosclerosis", “carotid artery diseases”, “cardiovascular diseases”, “cerebrovascular disease”, “cerebrovascular disorders”, “coronary artery disease”, “heart diseases”, “hypertension”, “infarct”, “ischemia”, “ischemic heart disease”, “myocardial infarction”, “peripheral vascular diseases”, “peripheral arterial disease”, “stroke” and were combined with “arsenic”, “arsenic poisoning”, “arsenicals”, “arsenite” and “arsenate”. The terms were combined with the Boolean operators "OR" or "AND". The reference lists and cross-references of eligible studies were also searched as were the bibliographies of recent systematic reviews and meta-analyses reported by Navas-Acien et al. [33], Phung et al. [34], Moon et al. [24], Tsuji et al. [22], Abhyankar et al. [35] and Chowdhury et al. [40].

Studies not published in English, related to specific scenario like childhood exposure or exposure during pregnancy or occupational exposure, or based on a specific form of As like arsenic trioxide (which is not the common form of As exposure in the general population) were excluded. The review of literature was conducted in accordance with the techniques specified by Stroup et al. [41] for meta-analysis of observational studies in epidemiology (MOOSE) and reporting was done based on Preferred Reporting Items for Systematic Reviews and Meta-Analyses (PRISMA) criteria [32].

Based on the abstract review, studies were firstly excluded if (a) they were case reports or case series; (b) no original data were presented; (c) had no human records; or (d) the outcome measures were not related with mortality or morbidity risk of relevant CVD types and/or clinical indicators considered in this study. The full text of studies which was regarded as potentially eligible was then assessed and further excluded if they were (a) not related with dietary As exposure or not for the general population; (b) no available relative risk (risk ratios, odds ratios) and measures of variability of different types of CVD were mentioned; or c) no categorical As exposure (at least three categories) was used (Figure S1). The methodological quality of the studies was also assessed and those with missing data on, or incomplete definitions of, the study design, population, exposure condition, or outcome variables were excluded [42,43,44,45,46,47,48,49]. Furthermore, as it is difficult to convert plasma As to water As, we excluded two studies which could only provide plasma As concentration to lower the bias of exposure assessment [50,51]. In addition, as there was only one study analysing each of soluble E-selectin, myeloperoxidase, plasminogen activator inhibitor-1 (PAI-1), soluble Intercellular Adhesion Molecule 1 (ICAM-1) and soluble Vascular cell adhesion protein 1 (VCAM-1) [18], carotid atherosclerosis indices (CAIs) [52], common carotid intima media thickness, plaque score and the presence of plaque in the common carotid [53], peripheral vascular disease [54,55] as well as some other CVD subtypes and clinic markers [21,27,56,57,58,59,60,61], respectively, these variables have not been included in our dose-response meta-analysis. Moreover, due to the fact that significant differences could be found regarding concentrations of matrix-metalloproteases (MMPs) using plasma vs. serum [62], we further excluded two studies which analysed the plasma MMPs and serum MMPs respectively to avoid bias in outcome ascertainment [18,21] (Figure S1). Two reviewers (LX and DM) independently reviewed the quality of the studies and differences were resolved by consensus and discussion.

### 2.2. Data Extraction

For each study that appeared to meet the inclusion criteria, we extracted data on the number of deaths or cases, person-years or total number of population, the measured risk values (e.g., rate ratio, hazard ratio, odds ratio) and their statistical uncertainty (95% confidence interval (CI)) for each As concentration category. In addition, we recorded the descriptive information including authors’ names, year of study publication, sampling method, sample size, study design, and outcome ascertainment as well as the adjustment for confounders.

Similar to the method used by Moon et al. [24], we combined all measures of association (e.g., rate ratio, hazard ratio, odds ratio) in different studies for each CVD endpoint together, as the relative risk. For the exposure assessment, we abstracted the mean, median values, and the range of As concentration in each concentration category. Though drinking water was the main metric of As exposure, for studies where drinking water data was not directly available, we estimated the drinking water As concentration from either toenail As [19] using the following formulae (see Equations (1) and (2)):

(1) water As (µg/L) ≥ 1 (µg/L)=10^(1.4+0.9×log10(toenail As, µg/kg)),

(2) water As (µg/L) < 1 (µg/L)=10^(−0.4+0.9×log10(toenail As, µg/kg)),

or from urinary As [63] using Equation (3):

(3) water As (µg/L)=urinary As (µg/g creatinine)×mean urine creatinine (g/L),

where it was assumed that each participant has a urine creatinine concentration equal to the overall mean in the Strong Heart Study baseline visit (1.3 g/L, Moon KA, unpublished data). The credibility about the estimation of drinking water As concentration from toenail As and urinary As is indicated by their successful use in previous meta-analysis studies such as those of Moon et al. [24].

### 2.3. Statistical Analysis

A random-effects dose-response meta-analysis was conducted to estimate the pooled dose-response relationship between the log-transformed relative risk of each CVD outcome and water As concentrations from the summarized data of multiple studies using the package ‘dosresmeta’ in R statistical software [64,65]. This ‘dosresmeta’ meta-analysis consists of a two-stage procedure, whereby the study-specific trends are firstly estimated and then pooled across studies. In this analysis, we assumed both a constant log-linear (log-transformed water As concentration) and a flexible non-linear (restricted cubic splines with knots at the 10th, 50th and 90th percentiles of log-transformed water As concentration) associations. The median value was assigned as the concentration level for each concentration category and if the median value was not provided directly, the mean or the midpoint values were calculated instead [16,25,45,59,63,66,67,68,69,70,71,72,73,74,75]. For the calculation of the midpoint value for each concentration category, zero was used as the minimum if not available [16,25,66,68,69,70,71,72,73]. Furthermore, without available maxima values, the width of the highest concentration category was assumed to be equal to the next lowest category [16,25,66,68,69,70,71,72,73]. Pooled log-linear and non-linear relative risks for each CVD endpoint were estimated for water As concentration of 3 µg/L, 5 µg/L, 10 µg/L, 20 µg/L and 50 µg/L, using 1 µg/L of drinking water As as the reference exposure.

Dose-response relationships for individual studies with at least three concentration categories were plotted using the ‘ggplot’ function in the ‘ggplot2’ package in R and the pooled dose-response relationship for each CVD endpoint was plotted using the ‘matplot’ function in R to visualize the predicted relative risks at higher As level compared with the referent concentration (1 µg/L).

Forest plots were used to show the pooled relative risks (95% CI) for both individual studies and the overall one, comparing risk for chronic exposure to drinking water As of 10 µg/L with 1 µg/L using the ‘forestplot’ function of the ‘metafor’ package in R, with the sizes of the squares of individual study relative risks weighted by the inverse variance of the log-relative risk within each model. Specifically, both the individual and the overall pooled relative risks (95% CIs) in the forest plots were calculated via the dose-response meta-analysis through the ‘dosresmeta’ package in R. Model goodness-of-fit was assessed using deviance tests, coefficient of determination (R^2^ and adjusted R^2^) and Akaike’s information criterion (AIC) values via the ‘gof’ function in the ‘dosresmeta’ package in R. Furthermore, dose-response relationships for individual studies were overprinted by the pooled dose-response relationship for each CVD endpoint to visually test the model goodness-of-fit. Heterogeneity was assessed by P-heterogeneity (*p* < 0.05 being significant), I^2^-statistics (I^2^-statistic < 25% indicating low heterogeneity, 25%–50% moderate, and > 50% high) and Cochran’s Q-statistic [76].

In addition, to examine the potential publication bias and small study effects, funnel plots for all the CVD endpoints and Egger’s test of funnel plot asymmetry for those with greater than two studies were created using the ‘metafor’ package in R. To be specific, the effect estimated from each study (log-relative risk) was plotted against the standard error of log-relative risk, with the funnel centred at the overall model estimate.

Finally, sensitivity analyses were conducted. On one hand, to test the reliability and suitability of combining different exposure media, we conducted sensitivity analysis to estimate the relationships between CVD risks and drinking water As both linearly and non-linearly by excluding studies which did not provide water As concentrations directly. On the other hand, with the intention of analysing the effects of low-level As exposure, we also excluded studies with As concentration higher than 100 µg/L and conducted subgroup analysis only for low to moderate As concentrations (taken here to be < 100 µg/L), modelling its linear and non-linear associations with the CVD risks.

All our data analysis was performed using R statistical software (R Foundation for Statistical Computing, Vienna, Austria), version 3.4.3 (R Foundation for Statistical Computing) and R Studio (R Studio Desktop 1.1.423) [65,77,78].

## 3. Results

### 3.1. Study Characteristics

For our systematic review and meta-analysis, we identified 28 studies which satisfied the inclusion criteria (Table S1). There were considerable variations across studies in terms of the study design, study areas, exposure assessment, methods of outcome ascertainment and adjustment for confounders.

Among all these studies, there were 13 cohort studies [15,17,19,20,26,29,63,71,79,80,81,82], with one retrospective study [57], inferring causality between CVD endpoints and As exposure; six cross-sectional studies [59,66,67,68,70,75]; five case-control studies [16,69,72,73,83]; one ecological study [74]; and three case-cohort studies [20,36,84].

We have included studies from As-endemic areas, such as Bangladesh [15,17,20,67,70], China [66,68,79,83], Taiwan [69,71,72,81,82], Chile [16] and Mexico [59] where recorded As level in drinking water could be as high as 900 µg/L, and studies from what are generally considered as non-endemic areas such as parts of North America [19,63,84] and some European countries [29,74].

We observed substantial differences in the methods of exposure assessment across studies (Table S1): while most provided As concentrations directly in drinking water [17,20,67,68,69,72,73,74,79,80,81,82,84], some studies also reported exposure based on urine [26,29,36,63,66] or toenails [19]. In addition, although many studies analysed As exposure at the individual or household-level [59,73,80], some studies tested As exposure at the municipal- or village-level instead [74] and may have greater measurement error. Moreover, while several studies applied time-weighted average [17,29,70,80,84] or accumulative average [16,66,67,69,70,71,72], many studies still used As concentration analysed at one time point as a proxy for chronic exposure [15,20,68,73].

In this study, the different CVD outcomes considered were: carotid atherosclerosis [69,72,73], CHD [15,19,20,29,63,74,79,81,83,84], overall CVD [15,19,20,29,63,74,79,80,82], hypertension [16,26,59,66,67,70,71,75], stroke [15,17,19,20,29,36,57,63,74,79] and the CVD clinic markers considered were: QT prolongation [68,85] and pulse blood pressure [67,75]. There were considerable differences in the outcome ascertainment of these CVD endpoints with quality of those ascertainment methods varying across studies (Additional file 1: Table S1). On one hand, some researchers confirmed the outcomes through standard clinical practice or self-reporting information, for example, hypertension risk was identified via blood pressure tests by trained clinicians using a sphygmomanometer or self-reporting information from either a physician’s diagnosis or the record of anti-hypertensive medication usage [16,26,66,70]. On the other hand, some outcomes were identified by reviewing relevant database or records, ranging from the US National Death Index [19], National Institute for Statistics [74] to National Death Registry in Taiwan [81,82] and some other hospitalization and death records [63].

Association between CVD risks and As exposure could vary by age, gender, smoking status, alcohol consumption, physical activity, diabetes and obesity status, education level as well as some other socio-economic factors, all of which might be either powerful predictors of CVD risks or important factors in As toxicity [86,87,88,89,90,91,92,93,94]. However, varied adjustment for these well-identified potential confounders could be observed among different studies, leading to different magnitudes of associations estimated when combined in a meta-analysis. All the studies were adjusted for at least age and gender, while BMI [15,20,63,83,84], education level [15], and smoking status [19,20,63,81] were adjusted in some, and few studies had some other health and socio-economic indicators [29,63,74,81] adjusted for. Moon et al. [63], and Wade et al. [79] even adjusted for some more rarely used variables, notably estimated glomerular filtration rate, albuminuria and farm work.

### 3.2. Pooled Association between As Level and CVD Risk

Of the 28 selected studies, 22 studies included dose-response meta-analysis of mortality risk, of which there were seven for CHD, eight for CVD and seven for stroke. The 21 studies on combined fatal and non-fatal CVD risks included three studies on carotid atherosclerosis disease, four studies on CHD, two studies on CVD, eight studies on hypertension and four studies on stroke. Apart from those quantifying the risk of different CVD types, we also considered four studies for CVD clinic markers, including two studies for pulse blood pressure and two studies for QT prolongation (Table 1).

**Figure 1:**
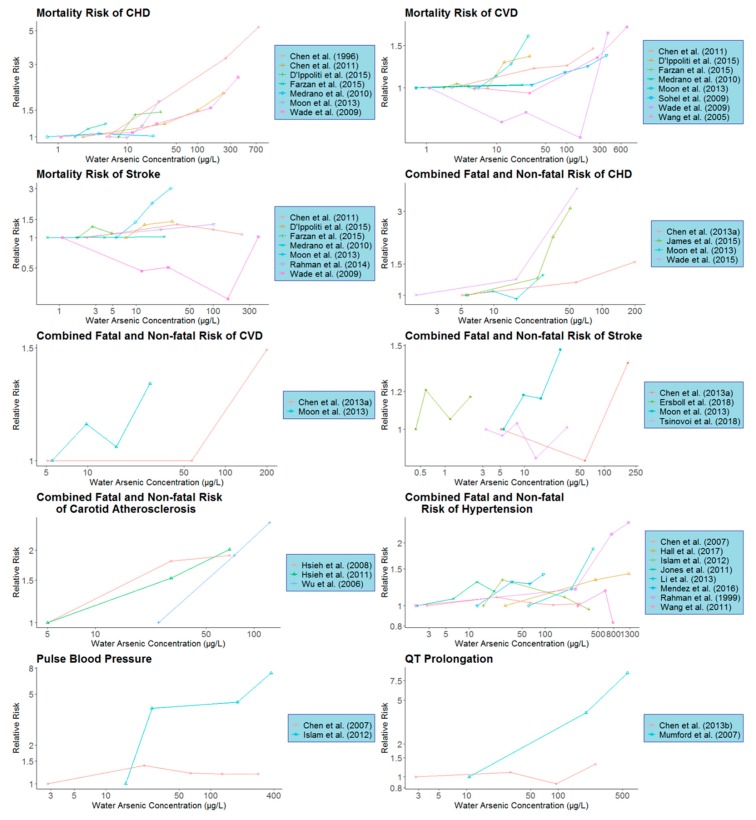
Individual study dose-response characteristics for various CVD subtypes or biomarkers. Arsenic concentrations refer to the observed or estimated median arsenic concentrations for the given concentration category. Lines connect the dose-response data for each study and are for illustrative purposes only (CVD: cardiovascular disease; CHD: coronary heart disease).

**Figure 2:**
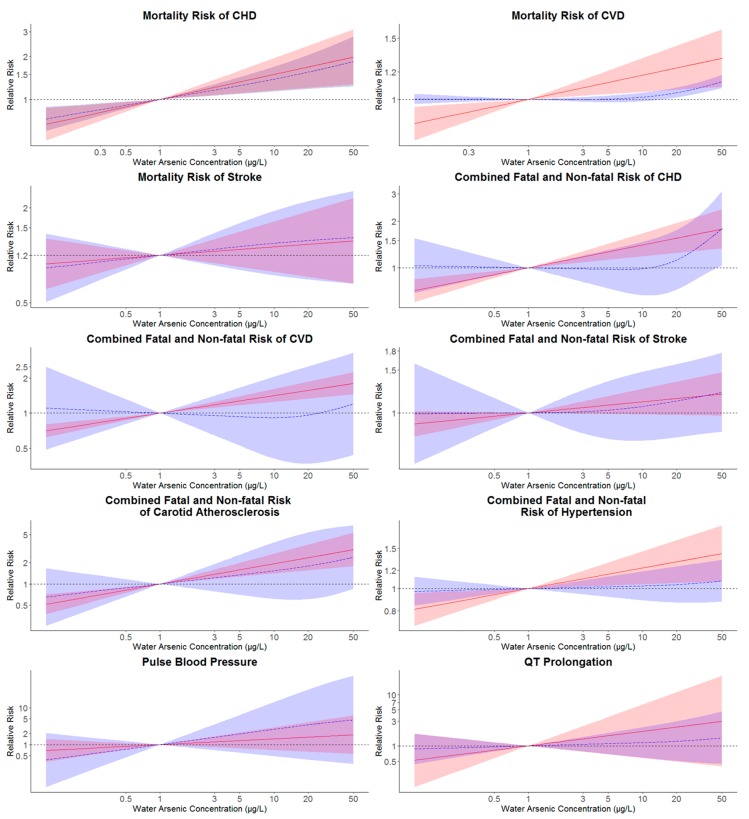
Pooled log-linear and non-linear relative risks and 95% confidence intervals (CIs) of different CVD endpoints in relation to the estimated drinking water arsenic concentration. Pooled log-linear and non-linear relative risks of CVD endpoints were estimated for drinking water arsenic concentrations with reference to an arsenic concentration of 1 µg/L. Solid lines (red) correspond to pooled relative risks of linear models with their 95% CIs represented as shaded regions (red). Pooled relative risks of non-linear models were represented by long-dash lines (blue) and their 95% CIs were plotted as shaded areas (blue). Log-linear models were estimated with log-transformed estimated drinking water arsenic concentration and non-linear associations were estimated from models with restricted cubic splines of log-transformed water arsenic concentration with knots at the 10th, 50th and 90th percentiles of log-transformed water arsenic (CVD: cardiovascular disease; CHD: coronary heart disease).

Individual dose-response information of all the studies included in our dose-response meta-analysis is presented in Table 1 and Figure 1. In the linear model, there was a significant overall trend for the mortality risk of CHD and CVD with an increase in As concentration (*p* < 0.05) (Table 2, Figure 2). Compared to 1 µg/L as the reference level, the relative mortality risk of CHD and CVD at 10 µg/L drinking water As concentration was 1.498 (95% CI: 1.153–1.948) and 1.174 (95% CI: 1.049–1.313), respectively (Table 2). Similarly, significantly overall trend (*p* < 0.05) was also obtained for the combined fatal and non-fatal risk of CHD, CVD, carotid atherosclerosis disease and hypertension with the relative risks of these four endpoints at 10 µg/L of water As concentration were 1.405 (95% CI: 1.183–1.667), 1.411 (95% CI: 1.242–1.603), 1.936 (95% CI: 1.403–2.671) and 1.231 (95% CI: 1.043–1.452), respectively, compared with 1 µg/L as the reference (Table 2 and Figure 2).

In the non-linear analysis, though the overall trend was significant (*p* < 0.05) for CHD and CVD mortality risks as well as the combined fatal and non-fatal risk of CHD and carotid atherosclerosis disease, significant increases in pooled relative risks at 10 µg/L drinking water As compared with 1 µg/L as a reference were only found for the mortality risk of CHD (relative risk: 1.387; 95% CI: 1.135–1.695) (Table 2 and Figure 2).

### 3.3. Heterogeneity

**Figure 3:**
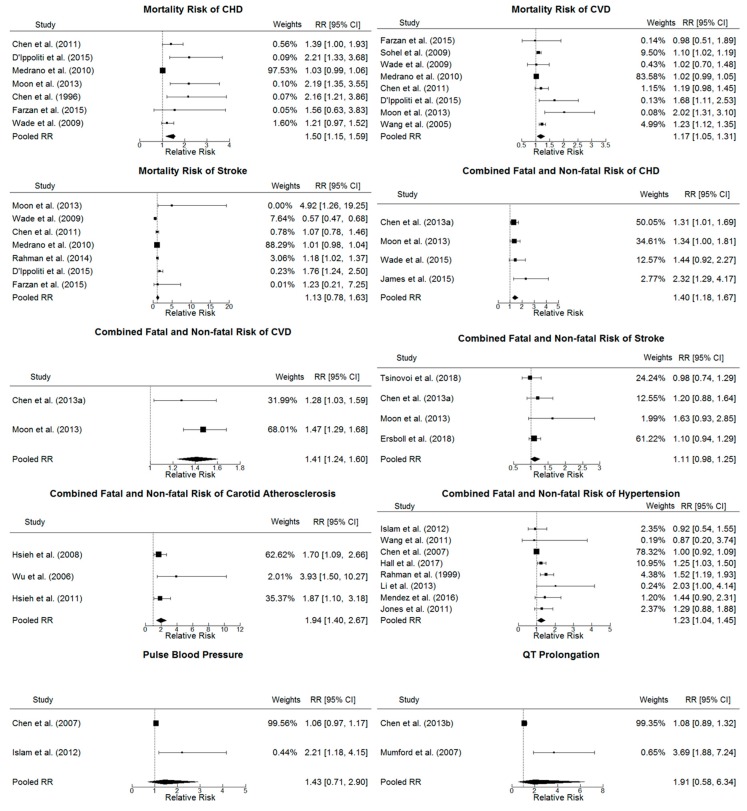
Forest plot of individual study and pooled log-linear relative risks (95% confidence intervals (CIs)) of different CVD endpoints, comparing 10 µg/L with 1 µg/L drinking water arsenic concentration. The sizes of squares of the individual study relative risks were weighted by the inverse variance of the log-relative risk within each model (CVD: cardiovascular disease; CHD: coronary heart disease)**.**

Combined with the estimated I^2^ statistic, Cochran’s Q-statistic and *p*-values for heterogeneity as presented in Table 2 and the individual and pooled relative risks of different CVD endpoints at 10 µg/L drinking water As in comparison with 1 µg/L shown in Figure 3, we found evidence of significantly high heterogeneity among the studies combined for the linear and non-linear analysis of the mortality risk of CHD, stroke and two CVD clinic markers (pulse blood pressure and QT prolongation) as well as the linear analysis of CVD mortality risk (heterogeneity *p*-values < 0.05 and I^2^ > 50%).

### 3.4. Model Goodness Assessment

Deviance, *p*-value, R^2^, adjusted R^2^ and AIC, as well as the difference between dose-response relationships for individual studies and the pooled dose-response relationship for each CVD endpoint, have been used for the goodness-of-fit assessment (Table 3 and Figure S2). According to the results, except for the linear analysis of pulse blood pressure (*p*-values lower than 0.05), deviance tests indicated no overall lack of fit for other CVD endpoints. In addition, there was only a slight increase in the R^2^ and the adjusted R^2^ for non-linear models compared with linear ones for most CVD endpoints, indicating that non-linear models explained more the variation of CVD risks. Linear models are considered a better fit for all the CVD endpoints due to their lower AIC values.

### 3.5. Small-study Effects Evaluation

Funnel plots and Egger’s test of funnel plots asymmetry suggested potential bias for the mortality risk of CHD and CVD with their *p*-values for Egger’s Regression Test lower than 0.05 (Table S2 and Figure S3).

### 3.6. Sensitivity Analysis

Excluding studies which cannot provide drinking water As concentrations directly resulted in similar conclusions to the main test, with the relative risks for both pooled linear and non-linear models becoming lower but with heterogeneity remaining high (Table S3).

Excluding studies with drinking water As concentration > 100 µg/L, the estimated pooled relative risks were higher for both pooled linear and non-linear models but heterogeneity was not reduced (Table S4).

## 4. Discussion

To our knowledge, this is the first dose-response meta-analysis estimating CVD risks from low-level As exposure from drinking water with As concentrations lower than the WHO provisional guideline value of 10 µg/L, to which a large population is exposed globally [95]. In this study, we found a significant increase of 49.8 % and 17.4% of CHD and CVD mortality risks, as well as 40.5%, 41.1%, 93.6% and 23.1% increased risk for combined fatal and non-fatal CHD, CVD, carotid atherosclerosis disease and hypertension at a drinking water As concentration of 10 µg/L compared to 1 µg/L based on the linear model, which is preferred over the non-linear model. Considering the sizable population exposed to such low-level As concentrations [95] and the high morbidity and mortality of CVD worldwide [96], such a proportional increase in CVD risks due to low-level drinking water As may lead to hundreds of thousands of additional CVD cases or even premature deaths, causing a serious cardiovascular health issue for people and health services around the world.

Already, strong evidence of the relationships between As exposure and different CVD endpoints has been identified in several systematic reviews and meta-analysis. However, most reviews were either related with a higher level of As exposure (> 50 µg/L) [22,28,33,40,97] or only provided descriptive evaluations [23,28]. To our knowledge, only Moon et al. [24] dose-response meta-analysis was available on this subject, where 11 individual studies were integrated to evaluate linear and non-linear associations between CVD incident risks and low-moderate water As. Compared to Moon et al. [24], our present analysis included not only several CVD types, but also two CVD clinical markers (QT prolongation and pulse blood pressure), providing a more comprehensive view of the possible effects of low-level As exposure. In addition, Moon et al. [24] estimated CVD relative risk by comparing 20 µg/L with 10 µg/L water As concentration, while in our study, we determined the relative risk at 10 µg/L and below, using 1 µg/L as the referent, addressing the gap, at least partly, on the CVD risks from low-level As exposure especially for As concentrations lower than the WHO provisional guideline value, to which a large population is exposed worldwide [95].

To reduce model misspecification, we conducted both a constant linear and a smooth non-linear model (restricted cubic splines with knots at the 10th, 50th and 90th percentiles of log-transformed water As) via a two-stage ’dosresmeta’ function. Somewhat consistent with previous meta-analysis researches [22,24], differences in the linear and non-linear model outcomes could be observed; notably there was no significant increased risk at 10 µg/L As for any of the outcomes except for CHD mortality risk in the non-linear model. Such a phenomenon might be related to the small number of studies and relatively few exposure categories in each study, which may be underpowered to establish precise dose-response relationships between CVD risks and low As exposure. It was particularly the case for the combined fatal and non-fatal risk of CVD and two CVD markers, where only two studies were found for each of these outcomes.

Importantly, our study could provide guidelines for future modelling assessment in epidemiological studies. We found the linear model better using AIC as the selection criteria depicting the associations of low-level As concentration and CVD outcomes. Such a conclusion has already been confirmed by several epidemiological and biological studies [36,53,61]. However, it has been illustrated that several mechanisms of As toxicity, ranging from the generation of oxidative stress, inhibition of DNA repair to modulation of signal transduction pathways and perturbation of DNA methylation patterns, are likely to give rise to nonlinear dose-response relationships, especially at low concentrations [98,99]. Besides, one recent study also revealed a steeper increase at lower As concentrations for fatal CVD [29]. Considering our limited sample size which may prevent discerning statistical evidence of a departure from a constant linear dose-response association and the significantly non-linear trends detected for some CVD endpoints (*p*-value for non-linear trend lower than 0.05); it is, therefore, important to bear in mind that the non-linear analysis of As effects should not be neglected, calling for future individual-level studies with sufficient data on low level As exposure and adverse health effects.

Unfortunately, our study also has several limitations, the most important of which might be the unavoidable heterogeneity in our meta-analysis. We assumed that the above-mentioned substantial differences in underlying population characteristics, adjustment for confounding, methods for outcome ascertainment and exposure assessment might be responsible for such heterogeneity. It has been well-documented that assessing As exposure via a biomarker of internal dose is of great importance as it could provide an integrated measure of all sources of exposure, not only from drinking water, but also from diet, soil and air particles [100,101]. It is especially the case for areas with low to moderate drinking water As concentrations, where drinking water might not be the major exposure pathway [102,103]. Notwithstanding this, different biomarkers reflect different periods of As exposure. For example, as toenails normally grow about 0.75 mm per month [104], they could reflect a long-term exposure, even remaining constant for up to 6 years [105]. Similarly, As levels in hair could reflect exposure from the past several months [97]. In contrast, urine and blood are regarded as measuring recent As exposure for only several days or hours, respectively, being recommended as a more reliable method for examining short-term exposure patterns [6,106]. Besides, the relationships between As concentrations in internal biomarker (urine, toenail, hair or plasma) and drinking water still are not well understood, based not only on the magnitude, frequency and duration of environmental As exposure, but also on the individual variability associated to metabolism, body weight, urine dilution and toenail or hair characteristics [107,108,109]. Therefore, the pooled estimation with As concentrations from different exposure media in the present study may result in exposure misclassification, particularly at our low drinking water As concentrations at which both diet and water can contribute substantially to total As exposure. Nevertheless, some assumptions are necessary to pool studies with different exposure metrics and our sensitivity analysis excluding studies limited to biomarkers yielded similar conclusions.

It is also important to understand that even though several perspective studies, such as the ones conducted by D’Ippoliti et al. [29] and Ersboll et al. [57] examined As exposure for a period of more than 20 years, the follow-up periods was still too short for the analysis of such low-level exposures (< 10 µg/L). Thus, more studies with longer periods of study period are warranted to confirm this association. Our meta-analysis might also be underpowered because of the small number of studies and concentration categories included in each CVD endpoint, producing a challenge for distinguishing the sources of heterogeneity. Furthermore, there was a lack-of-fit for the linear analysis of pulse blood pressure in this study, requiring further model improvement. Overall, given the high prevalence of low As exposure from different sources worldwide, long-term prospective studies with sufficient and high quality outcomes and exposure assessment at the individual level are needed [28].

These findings shed light, at least in part, on low-level As exposure induced health risks, suggesting that a consideration of downward revision of the WHO provisional guide value may be indicated to protect better human health and to reduce the economic burden arising from CVD related outcomes, even in countries such as the UK, where relatively few individuals consume drinking water with more than 10 µg/L As [39], but over 20 million people consume drinking water with between 1 µg/L As and 10 µg/L As [38]. Future carefully designed larger-scale perspective studies with sufficient individual data of CVD risk and low level environmental As exposure from different sources are necessary to evaluate the association between As exposure and increases in CVD risk.

## 5. Conclusions

Our meta-analysis indicates an overall positive association between CVD risks and low level (1 µg/L to 10 µg/L) As concentration in drinking water. For the general population, those with chronic exposure to As in drinking water at the WHO provisional guideline value limit (10 µg/L) compared to a reference population with 1 µg/L As are modelled to have significant excess CHD (50% (95% CI: 15%–95%)) and CVD (17% (95% CI: 5%–31%)) mortality risks, as well as significant excess combined fata land non-fatal risks of CHD (41 % (95% CI: 18%–67%)), CVD (41% (95% CI: 24%–60%)), carotid atherosclerosis (94% (95% CI: 40%–167%)) and hypertension (23% (95% CI: 4%–45%)). Given the high mortality and morbidity of CVD risks in the world [96], such an increase in the relative risks related to low-level As exposure would give rise to a large absolute number of CVD patients and avoidable premature deaths, as well as a substantive increase in the financial burden on health service systems. Uncertainties and limitations of the available data and models indicate that further studies particularly those aiming to provide a deeper mechanistic understanding of the link between CVD and exposure to As are warranted, as well as a consideration of tightening the existing WHO provisional guideline value for arsenic in drinking water.

## Figures and Tables

**Table 1:** Characteristics of studies included for dose-response meta-analysis.

Study (year)	Design	Cases	Person or Person-years	Exposure Media	Concentration Category	Median	RR (95% CI)		
**Mortality**									
**CHD**									
Chen et al. [15] (2011)	ir	14	20,064	water (µg/L)	0.1–12.0	2.3	1 (referent)		
		16	19,109		12.1–62.0	34.0	1.22	0.56	2.65
		15	18,699		62.1–148.0	101.0	1.49	0.70	3.19
		26	19,380		148.1–864.0	237.0	1.94	0.99	3.84
D’Ippoliti et al. [29] (2015)	ir	684	771,860	water (µg/L)	< 10	7.4	1 (referent)		
		573	713,276		10–20	12.9	1.40	1.19	1.64
		1014	904,129		> 20	29.7	1.46	1.07	2.01
Medrano et al. [74] (2010)	ci	88,566	18,978,000	water (µg/L)	< 1	0.7	1 (referent)		
		19,709	4,803,000		1–10	3.9	1.05	1.01	1.10
		4725	1,011,000		> 10	23.3	1.02	0.96	1.08
Moon et al. [63] (2013)	ir	68	13,616	urine (µg/g creatinine)	< 5.8	4.2	1 (referent)		
		67	13,430		5.8–9.7	7.5	0.99	0.70	1.41
		87	12,720		9.8–15.7	12.4	1.18	0.83	1.69
		119	12,033		> 15.7	21.8	1.71	1.19	2.44
Chen et al. [81] (1996)	ir	4	2748	water (µg/L)	< 10	5	1 (referent)		
		5	1417		10–500	255	3.30	0.80	13.69
		16	4309		≥ 510	755	5.30	1.49	18.85
Wade et al. [79] (2009)	ir	44	14,636	water (µg/L)	0–5	1.1	1 (referent)		
		26	9047		5.1–20	11.8	1.07	0.64	1.78
		72	21,367		20.1–100	26.2	1.22	0.82	1.82
		17	3313		100.1–300	156.1	1.55	0.88	2.73
		2	249		Over 300	387.9	2.47	0.50	12.18
Farzan et al. [19] (2015)	ir	57	898	toenail (µg/g)	0.01–0.07	0.05	1 (referent)		
		51	852		0.07–0.11	0.09	1.13	0.77	1.67
		46	754		0.11–3.26	0.23	1.22	0.82	1.82
CVD									
Chen et al. [15] (2011)	ir	43	20,064	water (µg/L)	0.1–12.0	2.3	1 (referent)		
		51	19,109		12.1–62.0	34.0	1.21	0.80	1.84
		41	18,699		62.1–148.0	101.0	1.24	0.80	1.93
		63	19,380		148.1–864.0	237.0	1.46	0.96	2.20
Sohel et al. [80] (2009)	ir	147	114,068	water (µg/L)	< 10	0.7	1 (referent)		
		168	139,233		10–49	31.8	1.03	0.82	1.29
		463	365,496		50–149	95.0	1.16	0.96	1.40
		318	241,930		150–299	201.2	1.23	1.01	1.51
		115	78,786		> 300	371.5	1.37	1.07	1.77
D’Ippoliti et al. [29] (2015)	ir	2752	771,860	water (µg/L)	< 10	7.4	1 (referent)		
		2115	713,276		10–20	12.9	1.28	1.08	1.51
		3514	904,129		> 20	29.7	1.36	1.06	1.74
Medrano et al. [74] (2010)	ci	285,049	18,978,000	water (µg/L)	< 1	0.7	1 (referent)		
		62,739	4,803,000		1–10	3.9	1.02	0.99	1.06
		13,962	1,011,000		> 10	23.3	1.03	0.98	1.08
Moon et al. [63] (2013)	ir	86	13,616	urine (µg/g creatinine)	< 5.8	4.2	1 (referent)		
		95	13,430		5.8–9.7	7.5	1.12	0.83	1.52
		115	12,720		9.8–15.7	12.4	1.26	0.92	1.73
		143	12,033		> 15.7	21.8	1.65	1.20	2.27
Wade et al. [79] (2009)	ir	97	14,636	water (µg/L)	0–5	1.1	1 (referent)		
		42	9047		5.1–20	11.8	0.72	0.32	1.60
		113	21,367		20.1–100	26.2	0.79	0.34	1.86
		24	3313		100.1–300	156.1	0.62	0.10	3.70
		3	249		Over 300	387.9	1.70	0.51	5.72
Farzan et al. [19] (2015)	ir	125	1987	toenail (µg/g)	0.01–0.07	0.05	1 (referent)		
		103	1691		0.07–0.11	0.09	1.04	0.80	1.35
		84	1334		0.11–3.26	0.23	0.99	0.74	1.32
Wang, et al. [82] (2005)	ir	428	19,360	water (µg/L)	<10	5.0	1 (referent)		
		84	2130		10–49	29.5	0.95	0.74	1.21
		116	2317		50–499	274.5	1.34	1.08	1.66
		60	1165		≥500	724.5	1.80	1.36	2.38
Stroke									
D’Ippoliti et al. [29] (2015)	ir	660	771,860	water (µg/L)	< 10	7.4	1 (referent)		
		448	713,276		10–20	12.9	1.33	1.12	1.58
		789	904,129		> 20	29.7	1.44	1.16	1.78
Farzan et al. [19] (2015)	ir	15	233	toenail (µg/g)	0.01–0.07	0.05	1 (referent)		
		16	243		0.07–0.11	0.09	1.28	0.64	2.61
		12	161		0.11–3.26	0.23	1.10	0.50	2.40
Rahman et al. [17] (2014)	ir	62	38,198	water (µg/L)	< 10	1.7	1 (referent)		
		196	156,362		10–49	21.1	1.20	0.92	1.57
		271	42,579		> 50	102.2	1.35	1.04	1.75
Moon et al. [63] (2013)	ir	6	13,616	urine (µg/g creatinine)	< 5.8	4.2	1 (referent)		
		17	13,430		5.8–9.7	7.5	1.41	0.54	3.67
		13	12,720		9.8–15.7	12.4	2.16	0.77	6.09
		18	12,033		> 15.7	21.8	3.03	1.08	8.50
Chen et al. [15] (2011)	ir	43	20,064	water (µg/L)	0.1–12.0	2.3	1 (referent)		
		51	19,109		12.1–62.0	34.0	1.35	0.75	2.43
		41	18,699		62.1–148.0	101.0	1.20	0.63	2.27
		63	19,380		148.1–864.0	237.0	1.07	0.54	2.12
Wade et al. [79] (2009)	ir	53	14,636	water (µg/L)	0–5	1.1	1 (referent)		
		16	9047		5.1–20	11.8	0.47	0.27	0.84
		41	21,367		20.1–100	26.2	0.51	0.34	0.79
		7	3313		100.1–300	156.1	0.25	1.10	2.95
		1	249		Over 300	387.9	1.02	0.16	6.71
Medrano et al. [74] (2010)	ci	81,368	18,978,000	water (µg/L)	< 1	0.7	1 (referent)		
		18,327	4,803,000		1–10	3.9	1.00	0.99	1.05
		3895	1,011,000		> 10	23.3	1.02	0.95	1.09
Fatal and non-fatal									
Carotid atherosclerosis disease									
Wu et al. [69] (2006)	cc	25	64	water (µg/L)	≤ 50.00	25	1 (referent)		
		46	95		50.01–100.00	75	1.90	0.90	3.80
		89	183		≥ 100.01	125	2.60	1.30	5.00
Hsieh et al. [72] (2008)	cc	17	48	water (µg/L)	< 10	5	1 (referent)		
		23	61		10.1–50	30	1.80	1.00	3.20
		195	370		> 50	70	1.90	1.10	3.10
Hsieh et al. [73] (2011)	cc	24	55	water (µg/L)	< 10	5	1 (referent)		
		31	81		10.1–50.0	30	1.53	0.67	3.50
		325	720		> 50.0	70	2.01	1.05	3.85
CHD									
Wade et al. [83] (2015)	cc	168	305	water (µg/L)	< 10	1.9	1 (referent)		
		105	236		10–39	16.0	1.23	0.78	1.93
		11	26		> 40	58.6	4.05	1.10	14.99
Moon et al. [63] (2013)	ir	202	12,146	urine (µg/g creatinine)	< 5.8	4.2	1 (referent)		
		206	11,701		5.8–9.7	7.5	1.05	0.86	1.28
		197	11,305		9.8–15.7	12.4	0.95	0.77	1.19
		241	10,586		> 15.7	21.8	1.30	1.04	1.62
James et al. [84] (2015)	ir	58	4806	water (µg/L)	1–20	5.7	1 (referent)		
		18	1335		20–30	25.3	1.25	0.70	2.31
		16	534		30–45	35.1	2.14	1.22	3.98
		4	98		45–88	50.5	3.12	1.12	9.02
Chen et al. [20] (2013)	ir	61	2823	water (µg/L)	0.1–25	5.1	1 (referent)		
		72	2718		25.1–107	57.0	1.18	0.75	1.84
		75	2770		108–864	198.5	1.54	1.02	2.31
CVD									
Moon et al. [63] (2013)	ir	265	12,146	urine (µg/g creatinine)	< 5.8	4.2	1 (referent)		
		297	11,701		5.8–9.7	7.5	1.14	0.95	1.35
		291	11,305		9.8–15.7	12.4	1.05	0.87	1.26
		331	10,586		> 15.7	21.8	1.32	1.05	1.28
Chen et al. [20] (2013)	ir	114	2823	water (µg/L)	0.1–25	5.1	1 (referent)		
		120	2718		25.1–107	57.0	1.00	0.67	1.50
		132	2770		108–864	198.5	1.49	1.06	2.11
Hypertension									
Wang et al. [71] (2011)	ir	93	618	water (µg/L)	< 538	269	1 (referent)		
		103	721		538–700	619	1.18	0.60	2.34
		83	634		> 700	781	0.83	0.40	1.68
Jones et al. [26] (2011)	ir	418	952	urine (µg/L)	< 4.2	2.1	1 (referent)		
		451	1057		4.2 to 8.3	6.3	1.08	0.83	1.40
		446	1090		> 8.3 to 17.1	12.7	1.30	0.94	1.80
		446	1068		> 17.1	21.5	1.17	0.75	1.83
Chen et al. [75] (2007)	cc	289	2242	water (µg/L)	0.1–8.0	2.8	1 (referent)		
		274	2116		8.1–40.8	23.2	1.10	0.90	1.33
		273	2187		40.9–91.0	63.9	1.03	0.85	1.25
		259	2181		91.1–176.0	128.1	1.01	0.83	1.22
		265	2184		176.1–864.0	283.1	1.02	0.84	1.23
Islam et al. [67] (2012)	cc	22	291	water (µg/L)	10–22	15.5	1 (referent)		
		19	208		23–32	27.5	1.33	0.67	2.62
		13	252		33–261	180.0	1.10	0.49	2.44
		12	243		≥ 262	376.0	0.96	0.42	2.23
Li et al. [66] (2013)	cc	29	120	water (µg/L)	< 100	61.0	1 (referent)		
		30	119		100 to 350	223.8	1.20	0.63	2.29
		45	121		> 350	427.7	1.87	1.02	3.42
Mendez et al. [59] (2016)	cc	106	260	water (µg/L)	< 25.5	12.8	1 (referent)		
		106	260		25.5–47.9	36.7	1.30	0.84	2.00
		109	259		47.9–79.0	63.5	1.27	0.82	1.94
		118	259		≥ 79.0	94.6	1.41	0.91	2.17
Hall et al. [16] (2017)	cc	140	323	water (µg/L)	< 60	30.0	1 (referent)		
		246	482		60–859	459.5	1.33	0.98	1.79
		225	450		> 859	1258.5	1.42	1.04	1.92
Rahman et al. [70] (1999)	cc	9	114	water (µg/L)	< 0	2	1 (referent)		
		50	623		0–500	250	1.20	0.60	2.30
		93	576		500–1000	750	2.20	1.10	4.30
		55	282		> 1000	1250	2.50	1.20	4.90
Stroke									
Tsinovoi et al. [36] (2018)	ir	150	637	water (µg/L)	2.72–3.72	3.3	1 (referent)		
		138	622		4.75–5.88	5.3	0.97	0.73	1.30
		139	624		8.26–9.18	8.1	1.03	0.77	1.38
		119	606		11.99–16.72	13.9	0.87	0.64	1.18
		125	608		26.11–54.81	34.1	1.01	0.74	1.36
Moon et al. [63] (2013)	ir	55	12,146	urine (µg/g creatinine)	< 5.8	4.2	1 (referent)		
		75	11,701		5.8–9.7	7.5	1.18	0.82	1.69
		62	11,305		9.8–15.7	12.4	1.16	0.77	1.72
		72	10,586		> 15.7	21.8	1.47	0.97	2.21
Chen et al. [20] (2013)	ir	50	2823	water (µg/L)	0.1–25	5.1	1 (referent)		
		46	2718		25.1–107	57.0	0.86	0.49	1.51
		52	2770		108–864	198.5	1.38	0.84	2.27
Ersboll et al. [57] (2018)	ir	486	172,202	water (µg/L)	0.049–0.573	0.435	1 (referent)		
		657	180,891		0.573–0.760	0.584	1.21	1.07	1.36
		475	169,470		0.760–1.933	1.174	1.05	0.92	1.19
		577	173,856		1.933–25.34	2.109	1.17	1.04	1.32
CVD markers									
Pulse blood pressure (SBP-DBP ≥ 55 mmHg))									
Chen et al. [75] (2007)	cc	205	2242	water (µg/L)	0.1–8.0	2.8	1 (referent)		
		252	2116		8.1–40.8	23.2	1.39	1.14	1.71
		232	2187		40.9–91.0	63.9	1.21	0.99	1.49
		227	2181		91.1–176.0	128.1	1.19	0.97	1.45
		233	2184		176.1–864.0	283.1	1.19	0.97	1.46
Islam et al. [67] (2012)	cc	5	291	water (µg/L)	10–22	15.5	1 (referent)		
		10	208		23–32	27.5	3.87	1.22	12.2
		10	252		33–261	180.0	4.32	1.23	15.11
		16	243		≥ 262	376.0	7.32	2.18	24.60
QT prolongation									
Chen et al. [85] (2013)	ir	57	371	water (µg/L)	0.1–9	2.8	1 (referent)		
		63	369		9.5–57	30.0	1.10	0.74	1.63
		49	374		58–144	95.1	0.87	0.57	1.31
		68	353		145–790	254.5	1.31	0.87	1.96
Mumford et al. [68] (2007)	cc	4	103	water (µg/L)	< 21	10.7	1 (referent)		
		12	108		100–350	199.9	3.83	1.13	12.99
		21	102		430–690	568.3	8.85	2.72	28.75

CVD: cardiovascular disease; CHD: coronary heart disease. RR: Relative risk or approximation of the relative risk (rate ratio, risk ratio, odds ratio). ir: Risks estimated in the studies as rate ratio (incidence-rate data); ci: Risks estimated in the studies as risk ratio (cumulative incidence data); cc: Risks estimated in the studies as an odds ratio (see details reported by Orsini et al. [65]).

**Table 2:** Pooled relative risks (95% CIs) for different types of cardiovascular disease (CVD) and clinic markers in relation to water arsenic concentrations.

	Mortality Risk			Combined Fatal and Non-Fatal Risk					CVD Markers	
	CHD (7(25)) ^a^	CVD (8(31)) ^a^	Stroke (7(25)) ^a^	CHD (4(14)) ^a^	CVD (2(7)) ^a^	Stroke (4(16)) ^a^	Carotid Atherosclerosis Disease (3(9)) ^a^	Hypertension (8(30)) ^a^	Pulse Blood Pressure (2(9)) ^a^	QT Prolongation (2(7)) ^a^
Log-linear dose-response association model										
1 µg/L ^b^	1.000	1.000	1.000	1.000	1.000	1.000	1.000	1.000	1.000	1.000
3 µg/L	1.213 (1.070, 1.374)	1.079 (1.023, 1.139)	1.061 (0.891, 1.262)	1.176 (1.083, 1.276)	1.178 (1.108, 1.252)	1.051 (0.992, 1.114)	1.370 (1.175, 1.598)	1.104 (1.020, 1.195)	1.187 (0.848, 1.662)	1.363 (0.770, 2.414)
5 µg/L	1.327 (1.105, 1.593)	1.118 (1.034, 1.210)	1.090 (0.844, 1.407)	1.268 (1.125, 1.429)	1.272 (1.163, 1.391)	1.076 (0.989, 1.171)	1.587 (1.267, 1.987)	1.156 (1.030, 1.298)	1.286 (0.785, 2.105)	1.574 (0.682, 3.636)
10 µg/L	1.498 (1.153, 1.948)	1.174 (1.049, 1.313)	1.131 (0.784, 1.630)	1.405 (1.183, 1.667)	1.411 (1.242, 1.603)	1.111 (0.984, 1.254)	1.936 (1.403, 2.671)	1.231 (1.043, 1.452)	1.433 (0.707, 2.901)	1.914 (0.578, 6.339)
20 µg/L	1.693 (1.203, 2.380)	1.232 (1.064, 1.426)	1.173 (0.729, 1.889)	1.556 (1.245, 1.944)	1.566 (1.325, 1.848)	1.146 (0.979, 1.342)	2.362 (1.553, 3.590)	1.310 (1.057, 1.625)	1.597 (0.637, 3.998)	2.327 (0.490,11.052)
50 µg/L	1.988 (1.274, 3.103)	1.313 (1.085, 1.589)	1.233 (0.662, 2.295)	1.781 (1.331, 2.383)	1.796 (1.445, 2.230)	1.195 (0.973 1.469)	3.071 (1.777, 5.308)	1.423 (1.074, 1.885)	1.842 (0.555, 6.109)	3.012 (0.394, 23.045)
coefficient	0.1757	0.070	0.054	0.148	0.150	0.046	0.287	0.090	0.156	0.282
*p*-value for trend ^c^	0.003	0.005	0.510	< 0.001	< 0.001	0.090	< 0.001	0.014	0.320	0.290
I^2 d^	79.7%	77.9%	89.0%	6.6%	17.4%	0.0%	17.5%	62.3%	80.4%	91.5%
Cochran’s Q-statistic	29.54	31.70	54.78	3.21	1.21	2.88	2.43	18.56	5.10	11.7
P-heterogeneity ^e^	< 0.001	< 0.001	< 0.001	0.360	0.271	0.409	0.297	0.097	0.024	0.006
Non-linear dose-response association model (restricted cubic splines)										
1 µg/L ^b^	1.000	1.000	1.000	1.000	1.000	1.000	1.000	1.000	1.000	1.000
3 µg/L	1.163 (1.060, 1.276)	0.999 (0.983, 1.014)	1.092 (0.862, 1.382)	0.985 (0.811, 1.197)	0.954 (0.647, 1.406)	1.011 (0.823, 1.241)	1.225 (0.783, 1.917)	1.012 (0.944, 1.085)	1.578 (0.707, 3.523)	1.070 (0.772, 1.483)
5 µg/L	1.250 (1.090, 1.433)	1.001 (0.980, 1.023)	1.136 (0.807, 1.596)	0.978 (0.735, 1.302)	0.933 (0.528, 1.648)	1.026 (0.780, 1.349)	1.347 (0.699, 2.594)	1.018 (0.920, 1.128)	1.951 (0.601, 6.326)	1.105 (0.685, 1.781)
10 µg/L	1.387 (1.135, 1.695)	1.015 (0.986, 1.043)	1.192 (0.746, 1.902)	0.986 (0.663, 1.468)	0.915 (0.410, 2.040)	1.063 (0.766, 1.474)	1.537 (0.612, 3.863)	1.027 (0.888, 1.187)	2.601 (0.483, 14.001)	1.155 (0.583, 2.288)
20 µg/L	1.557 (1.182, 2.052)	1.045 (1.012, 1.080)	1.241 (0.701, 2.195)	1.124 (0.720, 1.754)	0.963 (0.371, 2.499)	1.120 (0.791, 1.586)	1.800 (0.605, 5.353)	1.041 (0.868, 1.249)	3.449 (0.389, 30.605)	1.229 (0.504, 2.996)
50 µg/L	1.846 (1.231, 2.769)	1.125 (1.077, 1.176)	1.295 (0.659, 2.542)	1.795 (1.029, 3.131)	1.199 (0.439, 3.273)	1.214 (0.834, 1.769)	2.394 (0.852, 6.728)	1.082 (0.877, 1.334)	4.642 (0.298, 72.343)	1.433 (0.440, 4.667)
*p*-value for trend ^f^	0.006	< 0.001	0.750	0.047	0.078	0.340	< 0.001	0.200	0.150	0.270
I^2 d^	69.8%	35.3%	80.0%	41.0%	53.7%	0.0%	0.0%	46.3%	73.1%	72.5%
Cochran’s Q-statistic	39.75	21.65	60.02	10.16	4.32	5.59	2.58	26.07	7.43	7.27
P-heterogeneity ^e^	< 0.001	0.086	< 0.001	0.117	0.115	0.470	0.629	0.025	0.024	0.026

CVD: cardiovascular disease; CHD: coronary heart disease. a: Sum of studies included; the total number of relative risks in each model. b: treat 1 µg/L water arsenic concentration as the referent. c: *p*-value for linear trend from a Wald test of the coefficient for drinking water arsenic concentrations. d: Proportion of total variance due to between-study heterogeneity. e: *p*-value for heterogeneity is chi-square *p*-value of the Q-statistic. f: Non-linear trend *p*-value for the non-linear spline coefficient in a model with arsenic concentrations entered as a restricted cubic spline with knots at 10th, 50th and 90th percentiles of water arsenic concentration.

**Table 3:** Goodness-of-fit assessment.

Studies	Mortality Risk			Combined Fatal and Non-fatal Risk					CVD Markers	
	CHD	CVD	Stroke	CHD	CVD	Stroke	Carotid Atherosclerosis Disease	Hypertension	Pulse Blood Pressure	QT Prolongation
Log-linear dose-response association model										
Deviance ^a^	19.40	22.58	15.98	13.04	7.06	18.54	2.99	20.27	14.02	4.97
Degrees of freedom ^b^	17	22	17	9	4	11	5	21	6	4
*p*-value ^c^	0.306	0.426	0.526	0.161	0.133	0.070	0.702	0.504	0.029	0.291
R^2^	0.320	0.258	0.027	0.537	0.798	0.134	0.844	0.230	0.066	0.185
Adjusted R^2^	0.280	0.225	−0.031	0.486	0.748	0.055	0.813	0.193	−0.089	−0.019
AIC	0.17	−6.77	6.58	−0.56	1.26	−2.22	3.38	−4.36	4.55	5.58
Non-linear dose-response association model (restricted cubic splines)										
Deviance ^a^	17.28	22.81	15.39	5.83	3.94	17.48	1.71	12.94	9.16	3.44
Degrees of freedom ^b^	16	21	16	8	3	10	4	20	5	3
*p*-value ^c^	0.367	0.354	0.496	0.666	0.267	0.064	0.789	0.880	0.103	0.328
R^2^	0.373	0.620	0.035	0.512	0.564	0.110	0.892	0.199	0.292	0.435
Adjusted R^2^	0.297	0.584	−0.085	0.390	0.273	−0.068	0.838	0.118	0.008	0.058
AIC	29.95	5.89	23.86	12.34	10.37	16.45	13.75	23.55	12.16	11.43

CVD: cardiovascular disease; CHD: coronary heart disease. a: Measure of the total absolute deviation between reported and predicted log-relative risk taking into account the covariance structure of the residuals. b: Degrees of freedom from the deviance statistic. c: *p*-value from test for model specification. AIC: Akaike’s information criterion.

## Supplementary Materials

The following are available online at https://www.mdpi.com/1660-4601/17/7/2536/s1, Table S1: Epidemiological studies of arsenic (As) exposure and cardiovascular disease (CVD) included in the systematic review, Table S2: Egger’s regression test of funnel plot asymmetry, Table S3: Pooled relative risks (95% confidence intervals) for different CVD types and clinical markers in relation to drinking water arsenic concentrations with the exclusion of studies which do provide drinking water As concentrations directly, Table S4: Pooled relative risks (95% confidence intervals) for different CVD types and CVD markers in relation to drinking water arsenic concentrations lower than 100 ppb, Figure S1: Flow diagram of study selection procedure, Figure S2: Association of CVD endpoints with drinking water arsenic concentrations, Figure S3: Funnel Plots for the analysis of publication bias.
