# Corrections: Xu, L.; Mondal, D.; Polya, D.A. Positive Association of Cardiovascular Disease (CVD) with Chronic Exposure to Drinking Water Arsenic (As) at Concentrations below the WHO Provisional Guideline Value: A Systematic Review and Meta-Analysis. *Int. J. Environ. Res. Public Health* 2020, *17*, 2536

**DOI:** 10.3390/ijerph17238947

**Published:** 2020-12-02

**Authors:** Lingqian Xu, Debapriya Mondal, David A. Polya

**Affiliations:** 1 Department of Earth and Environmental Sciences and Williamson Research Centre for Molecular Environmental Science, University of Manchester, Manchester M13 9PL, UK; lingqian.xu@postgrad.manchester.ac.uk (L.X.); david.polya@manchester.ac.uk (D.A.P.); 2 School of Science, Engineering & Environment, University of Salford, Salford M5 4WT, UK

In our recently published meta-analysis, due to an oversight, we treated urinary As concentration data reported by Tsinovoi et al. [1] instead as drinking water As data. This oversight impacted, in minor way, our linear and non-linear published dose-response models for combined fatal and non-fatal strokes. The oversight does not impact, in any way, any of our other published [2] dose-response models.

We corrected both Table 1 and Supplementary Materials Table S1; the exposure media for Tsinovoi et al. [1] is changed to ‘urinary As (µg/g creatinine)’ from ‘water As (µg/L)’. Accordingly, we modified dose-response models (Table 2) and goodness of fit parameters (Table 3) for the relationships between drinking water As and combined fatal and non-fatal risks of strokes in the corrected Manuscript [2]. These are based on using Equation (3) of Xu et al. [2] to calculate equivalent drinking water As concentrations from the reported urinary As values from Tsinovoi et al. [1]. Over the drinking water arsenic concentration range 1 to 50 µg/L, the absolute differences between the originally published and corrected relative risks (RR) for the linear and non-linear dose-response models for combined fatal and non-fatal stroke risks are all < 0.001 and < 0.020 respectively.

We also made the required corrections in Figure 1 and Figure 2 and Supplementary Figure S2, although these are almost identical to the original figures.

Lastly, we note that the corrections to the linear and non-linear dose-response models for combined fatal and non-fatal risks of strokes as a function of drinking water arsenic concentration show the same trends as in the original publication and, in particular, over the relatively low concentration range in the scope of the study, there remains no significant association in the data collated between drinking water As concentration and the combined fatal and non-fatal risks of stroke.

## Figures and Tables

**Figure 1:**
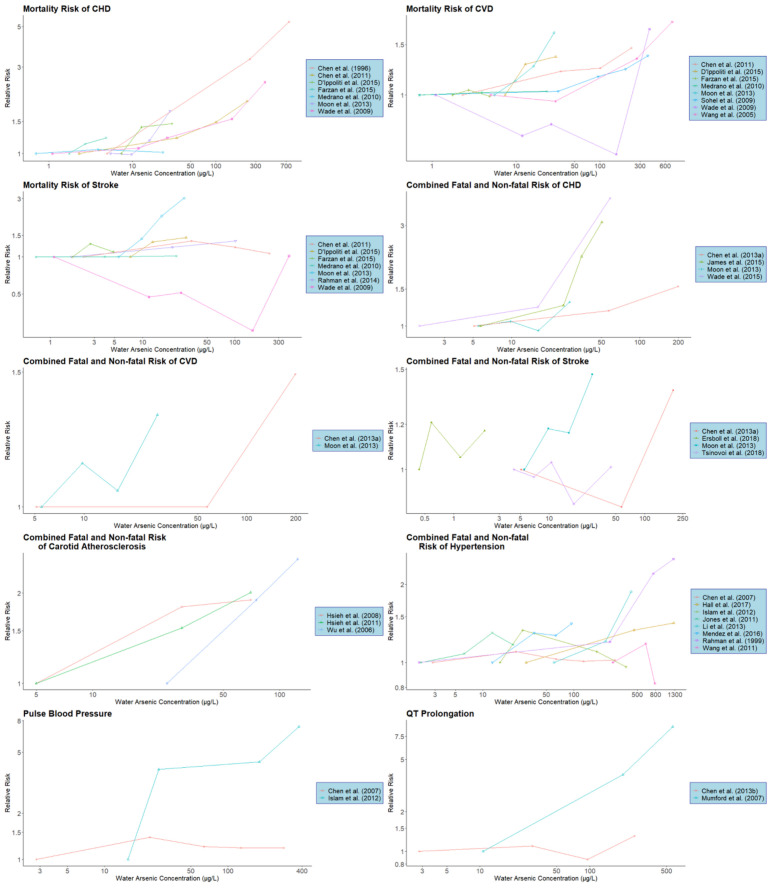
Individual study dose-response characteristics for various CVD subtypes or biomarkers. Arsenic concentrations refer to the observed or estimated median arsenic concentrations for the given concentration category. Lines connect the dose-response data for each study and are for illustrative purposes only (CVD: cardiovascular disease; CHD: coronary heart disease).

**Figure 2:**
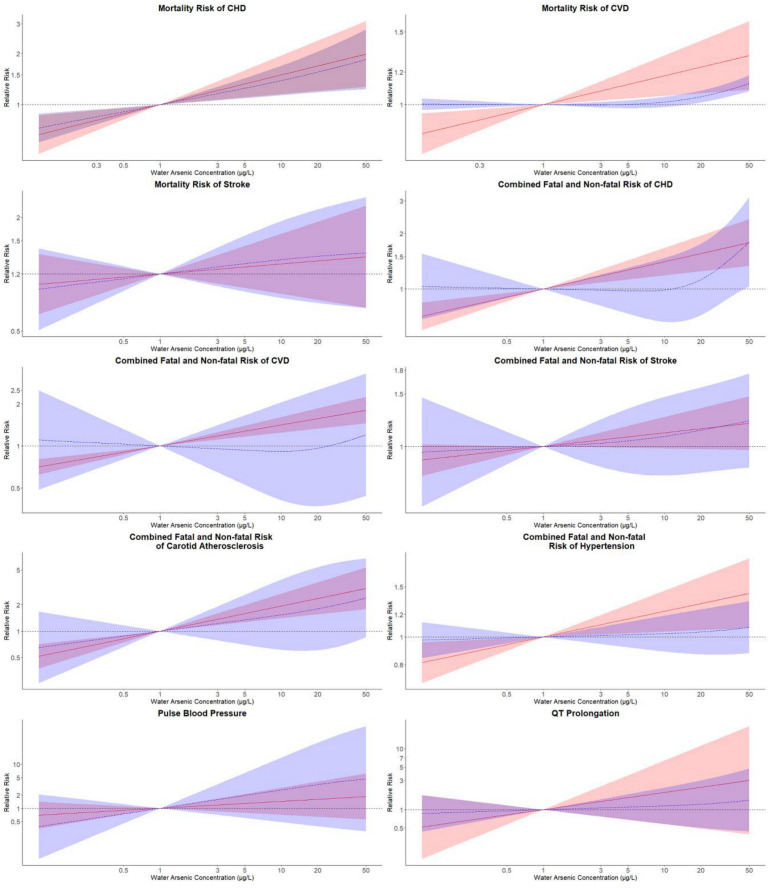
Pooled log-linear and non-linear relative risks and 95% confidence intervals (CIs) of different CVD endpoints in relation to the estimated drinking water arsenic concentration. Pooled log-linear and non-linear relative risks of CVD endpoints were estimated for drinking water arsenic concentrations with reference to an arsenic concentration of 1 µg/L. Solid lines (red) correspond to pooled relative risks of linear models with their 95% CIs represented as shaded regions (red). Pooled relative risks of non-linear models were represented by long-dash lines (blue) and their 95% CIs were plotted as shaded areas (blue). Log-linear models were estimated with log-transformed estimated drinking water arsenic concentration and non-linear associations were estimated from models with restricted cubic splines of log-transformed water arsenic concentration with knots at the 10th, 50th and 90th percentiles of log-transformed water arsenic (CVD: cardiovascular disease; CHD: coronary heart disease).

**Table 1:** Characteristics of studies included for dose-response meta-analysis.

Study (Year)	Design	Cases	Person or Person-Years	Exposure Media	Concentration Category	Median	RR (95% CI)		
**Mortality**									
**CHD**									
Chen et al. [15] (2011)	ir	14	20,064	water (µg/L)	0.1–12.0	2.3	1 (referent)		
		16	19,109		12.1–62.0	34.0	1.22	0.56	2.65
		15	18,699		62.1–148.0	101.0	1.49	0.70	3.19
		26	19,380		148.1–864.0	237.0	1.94	0.99	3.84
D’Ippoliti et al. [29] (2015)	ir	684	771,860	water (µg/L)	< 10	7.4	1 (referent)		
		573	713,276		10–20	12.9	1.40	1.19	1.64
		1014	904,129		> 20	29.7	1.46	1.07	2.01
Medrano et al. [74] (2010)	ci	88,566	18,978,000	water (µg/L)	< 1	0.7	1 (referent)		
		19,709	4,803,000		1–10	3.9	1.05	1.01	1.10
		4725	1,011,000		> 10	23.3	1.02	0.96	1.08
Moon et al. [63] (2013)	ir	68	13,616	urine (µg/g creatinine)	< 5.8	4.2	1 (referent)		
		67	13,430		5.8–9.7	7.5	0.99	0.70	1.41
		87	12,720		9.8–15.7	12.4	1.18	0.83	1.69
		119	12,033		> 15.7	21.8	1.71	1.19	2.44
Chen et al. [81] (1996)	ir	4	2748	water (µg/L)	< 10	5	1 (referent)		
		5	1417		10–500	255	3.30	0.80	13.69
		16	4309		≥ 510	755	5.30	1.49	18.85
Wade et al. [79] (2009)	ir	44	14,636	water (µg/L)	0–5	1.1	1 (referent)		
		26	9047		5.1–20	11.8	1.07	0.64	1.78
		72	21,367		20.1–100	26.2	1.22	0.82	1.82
		17	3313		100.1–300	156.1	1.55	0.88	2.73
		2	249		Over 300	387.9	2.47	0.50	12.18
Farzan et al. [19] (2015)	ir	57	898	toenail (µg/g)	0.01–0.07	0.05	1 (referent)		
		51	852		0.07–0.11	0.09	1.13	0.77	1.67
		46	754		0.11–3.26	0.23	1.22	0.82	1.82
CVD									
Chen et al. [15] (2011)	ir	43	20,064	water (µg/L)	0.1–12.0	2.3	1 (referent)		
		51	19,109		12.1–62.0	34.0	1.21	0.80	1.84
		41	18,699		62.1–148.0	101.0	1.24	0.80	1.93
		63	19,380		148.1–864.0	237.0	1.46	0.96	2.20
Sohel et al. [80] (2009)	ir	147	114,068	water (µg/L)	< 10	0.7	1 (referent)		
		168	139,233		10–49	31.8	1.03	0.82	1.29
		463	365,496		50–149	95.0	1.16	0.96	1.40
		318	241,930		150–299	201.2	1.23	1.01	1.51
		115	78,786		> 300	371.5	1.37	1.07	1.77
D’Ippoliti et al. [29] (2015)	ir	2752	771,860	water (µg/L)	< 10	7.4	1 (referent)		
		2115	713,276		10–20	12.9	1.28	1.08	1.51
		3514	904,129		> 20	29.7	1.36	1.06	1.74
Medrano et al. [74] (2010)	ci	285,049	18,978,000	water (µg/L)	< 1	0.7	1 (referent)		
		62,739	4,803,000		1–10	3.9	1.02	0.99	1.06
		13,962	1,011,000		> 10	23.3	1.03	0.98	1.08
Moon et al. [63] (2013)	ir	86	13,616	urine (µg/g creatinine)	< 5.8	4.2	1 (referent)		
		95	13,430		5.8–9.7	7.5	1.12	0.83	1.52
		115	12,720		9.8–15.7	12.4	1.26	0.92	1.73
		143	12,033		> 15.7	21.8	1.65	1.20	2.27
Wade et al. [79] (2009)	ir	97	14,636	water (µg/L)	0–5	1.1	1 (referent)		
		42	9047		5.1–20	11.8	0.72	0.32	1.60
		113	21,367		20.1–100	26.2	0.79	0.34	1.86
		24	3313		100.1–300	156.1	0.62	0.10	3.70
		3	249		Over 300	387.9	1.70	0.51	5.72
Farzan et al. [19] (2015)	ir	125	1987	toenail (µg/g)	0.01–0.07	0.05	1 (referent)		
		103	1691		0.07–0.11	0.09	1.04	0.80	1.35
		84	1334		0.11–3.26	0.23	0.99	0.74	1.32
Wang, et al. [82] (2005)	ir	428	19,360	water (µg/L)	<10	5.0	1 (referent)		
		84	2130		10–49	29.5	0.95	0.74	1.21
		116	2317		50–499	274.5	1.34	1.08	1.66
		60	1165		≥500	724.5	1.80	1.36	2.38
Stroke									
D’Ippoliti et al. [29] (2015)	ir	660	771,860	water (µg/L)	< 10	7.4	1 (referent)		
		448	713,276		10–20	12.9	1.33	1.12	1.58
		789	904,129		> 20	29.7	1.44	1.16	1.78
Farzan et al. [19] (2015)	ir	15	233	toenail (µg/g)	0.01–0.07	0.05	1 (referent)		
		16	243		0.07–0.11	0.09	1.28	0.64	2.61
		12	161		0.11–3.26	0.23	1.10	0.50	2.40
Rahman et al. [17] (2014)	ir	62	38,198	water (µg/L)	< 10	1.7	1 (referent)		
		196	156,362		10–49	21.1	1.20	0.92	1.57
		271	42,579		> 50	102.2	1.35	1.04	1.75
Moon et al. [63] (2013)	ir	6	13,616	urine (µg/g creatinine)	< 5.8	4.2	1 (referent)		
		17	13,430		5.8–9.7	7.5	1.41	0.54	3.67
		13	12,720		9.8–15.7	12.4	2.16	0.77	6.09
		18	12,033		> 15.7	21.8	3.03	1.08	8.50
Chen et al. [15] (2011)	ir	19	20,064	water (µg/L)	0.1–12.0	2.3	1 (referent)		
		26	19,109		12.1–62.0	34.0	1.35	0.75	2.43
		18	18,699		62.1–148.0	101.0	1.20	0.63	2.27
		22	19,380		148.1–864.0	237.0	1.07	0.54	2.12
Wade et al. [79] (2009)	ir	53	14,636	water (µg/L)	0–5	1.1	1 (referent)		
		16	9047		5.1–20	11.8	0.47	0.27	0.84
		41	21,367		20.1–100	26.2	0.51	0.34	0.79
		7	3313		100.1–300	156.1	0.25	1.10	2.95
		1	249		Over 300	387.9	1.02	0.16	6.71
Medrano et al. [74] (2010)	ci	81,368	18,978,000	water (µg/L)	< 1	0.7	1 (referent)		
		18,327	4,803,000		1–10	3.9	1.00	0.99	1.05
		3895	1,011,000		> 10	23.3	1.02	0.95	1.09
Fatal and non-fatal									
Carotid atherosclerosis disease									
Wu et al. [69] (2006)	cc	25	64	water (µg/L)	≤ 50.00	25	1 (referent)		
		46	95		50.01–100.00	75	1.90	0.90	3.80
		89	183		≥ 100.01	125	2.60	1.30	5.00
Hsieh et al. [72] (2008)	cc	17	48	water (µg/L)	< 10	5	1 (referent)		
		23	61		10.1–50	30	1.80	1.00	3.20
		195	370		> 50	70	1.90	1.10	3.10
Hsieh et al. [73] (2011)	cc	24	55	water (µg/L)	< 10	5	1 (referent)		
		31	81		10.1–50.0	30	1.53	0.67	3.50
		325	720		> 50.0	70	2.01	1.05	3.85
CHD									
Wade et al. [83] (2015)	cc	168	305	water (µg/L)	< 10	1.9	1 (referent)		
		105	236		10–39	16.0	1.23	0.78	1.93
		11	26		> 40	58.6	4.05	1.10	14.99
Moon et al. [63] (2013)	ir	202	12,146	urine (µg/g creatinine)	< 5.8	4.2	1 (referent)		
		206	11,701		5.8–9.7	7.5	1.05	0.86	1.28
		197	11,305		9.8–15.7	12.4	0.95	0.77	1.19
		241	10,586		> 15.7	21.8	1.30	1.04	1.62
James et al. [84] (2015)	ir	58	4806	water (µg/L)	1–20	5.7	1 (referent)		
		18	1335		20–30	25.3	1.25	0.70	2.31
		16	534		30–45	35.1	2.14	1.22	3.98
		4	98		45–88	50.5	3.12	1.12	9.02
Chen et al. [20] (2013)	ir	61	2823	water (µg/L)	0.1–25	5.1	1 (referent)		
		72	2718		25.1–107	57.0	1.18	0.75	1.84
		75	2770		108–864	198.5	1.54	1.02	2.31
CVD									
Moon et al. [63] (2013)	ir	265	12,146	urine (µg/g creatinine)	< 5.8	4.2	1 (referent)		
		297	11,701		5.8–9.7	7.5	1.14	0.95	1.35
		291	11,305		9.8–15.7	12.4	1.05	0.87	1.26
		331	10,586		> 15.7	21.8	1.32	1.05	1.28
Chen et al. [20] (2013)	ir	114	2823	water (µg/L)	0.1–25	5.1	1 (referent)		
		120	2718		25.1–107	57.0	1.00	0.67	1.50
		132	2770		108–864	198.5	1.49	1.06	2.11
Hypertension									
Wang et al. [71] (2011)	ir	93	618	water (µg/L)	< 538	269	1 (referent)		
		103	721		538–700	619	1.18	0.60	2.34
		83	634		> 700	781	0.83	0.40	1.68
Jones et al. [26] (2011)	ir	418	952	urine (µg/L)	< 4.2	2.1	1 (referent)		
		451	1057		4.2 to 8.3	6.3	1.08	0.83	1.40
		446	1090		> 8.3 to 17.1	12.7	1.30	0.94	1.80
		446	1068		> 17.1	21.5	1.17	0.75	1.83
Chen et al. [75] (2007)	cc	289	2242	water (µg/L)	0.1–8.0	2.8	1 (referent)		
		274	2116		8.1–40.8	23.2	1.10	0.90	1.33
		273	2187		40.9–91.0	63.9	1.03	0.85	1.25
		259	2181		91.1–176.0	128.1	1.01	0.83	1.22
		265	2184		176.1–864.0	283.1	1.02	0.84	1.23
Islam et al. [67] (2012)	cc	22	291	water (µg/L)	10–22	15.5	1 (referent)		
		19	208		23–32	27.5	1.33	0.67	2.62
		13	252		33–261	180.0	1.10	0.49	2.44
		12	243		≥ 262	376.0	0.96	0.42	2.23
Li et al. [66] (2013)	cc	29	120	water (µg/L-year)	< 100	61.0	1 (referent)		
		30	119		100 to 350	223.8	1.20	0.63	2.29
		45	121		> 350	427.7	1.87	1.02	3.42
Mendez et al. [59] (2016)	cc	106	260	water (µg/L)	< 25.5	12.8	1 (referent)		
		106	260		25.5–47.9	36.7	1.30	0.84	2.00
		109	259		47.9–79.0	63.5	1.27	0.82	1.94
		118	259		≥ 79.0	94.6	1.41	0.91	2.17
Hall et al. [16] (2017)	cc	140	323	water (µg/L)	< 60	30.0	1 (referent)		
		246	482		60–859	459.5	1.33	0.98	1.79
		225	450		> 859	1258.5	1.42	1.04	1.92
Rahman et al. [70] (1999)	cc	9	114	water (µg/L)	< 0	2	1 (referent)		
		50	623		0–500	250	1.20	0.60	2.30
		93	576		500–1000	750	2.20	1.10	4.30
		55	282		> 1000	1250	2.50	1.20	4.90
Stroke									
Tsinovoi et al. [36] (2018)	ir	150	637	urine (µg/g creatinine)	2.72–3.72	3.3	1 (referent)		
		138	622		4.75–5.88	5.3	0.97	0.73	1.30
		139	624		8.26–9.18	8.1	1.03	0.77	1.38
		119	606		11.99–16.72	13.9	0.87	0.64	1.18
		125	608		26.11–54.81	34.1	1.01	0.74	1.36
Moon et al. [63] (2013)	ir	55	12,146	urine (µg/g creatinine)	< 5.8	4.2	1 (referent)		
		75	11,701		5.8–9.7	7.5	1.18	0.82	1.69
		62	11,305		9.8–15.7	12.4	1.16	0.77	1.72
		72	10,586		> 15.7	21.8	1.47	0.97	2.21
Chen et al. [20] (2013)	ir	50	2823	water (µg/L)	0.1–25	5.1	1 (referent)		
		46	2718		25.1–107	57.0	0.86	0.49	1.51
		52	2770		108–864	198.5	1.38	0.84	2.27
Ersboll et al. [57] (2018)	ir	486	172,202	water (µg/L)	0.049–0.573	0.435	1 (referent)		
		657	180,891		0.573–0.760	0.584	1.21	1.07	1.36
		475	169,470		0.760–1.933	1.174	1.05	0.92	1.19
		577	173,856		1.933–25.34	2.109	1.17	1.04	1.32
CVD markers									
Pulse blood pressure (SBP-DBP ≥ 55 mmHg))									
Chen et al. [75] (2007)	cc	205	2242	water (µg/L)	0.1–8.0	2.8	1 (referent)						
		252	2116		8.1–40.8	23.2	1.39	1.14	1.71
		232	2187		40.9–91.0	63.9	1.21	0.99	1.49
		227	2181		91.1–176.0	128.1	1.19	0.97	1.45
		233	2184		176.1–864.0	283.1	1.19	0.97	1.46
Islam et al. [67] (2012)	cc	5	291	water (µg/L)	10–22	15.5	1 (referent)						
		10	208		23–32	27.5	3.87	1.22	12.2
		10	252		33–261	180.0	4.32	1.23	15.11
		16	243		≥ 262	376.0	7.32	2.18	24.60
QT prolongation									
Chen et al. [85] (2013)	ir	57	428	water (µg/L)	0.1–9	2.8	1 (referent)						
		63	432		9.5–57	30.0	1.10	0.74	1.63
		49	423		58–144	95.1	0.87	0.57	1.31
		68	421		145–790	254.5	1.31	0.87	1.96
Mumford et al. [68] (2007)	cc	4	103	water (µg/L)	< 21	10.7	1 (referent)						
		12	108		100–350	199.9	3.83	1.13	12.99
		21	102		430–690	568.3	8.85	2.72	28.75

CVD: cardiovascular disease; CHD: coronary heart disease. RR: Relative risk or approximation of the relative risk (rate ratio, risk ratio, odds ratio). ir: Risks estimated in the studies as rate ratio (incidence-rate data); ci: Risks estimated in the studies as risk ratio (cumulative incidence data); cc: Risks estimated in the studies as an odds ratio (see details reported by Orsini et al. [65]).

**Table 2:** Pooled relative risks (95% CIs) for different types of cardiovascular disease (CVD) and clinic markers in relation to water arsenic concentrations.

	Mortality Risk			Combined Fatal and non-Fatal Risk					CVD Markers	
	CHD (7(25)) ^a^	CVD (8(31)) ^a^	Stroke (7(25)) ^a^	CHD (4(14)) ^a^	CVD (2(7)) ^a^	Stroke (4(16)) ^a^	Carotid Atherosclerosis Disease (3(9)) ^a^	Hypertension (8(30)) ^a^	Pulse Blood Pressure (2(9)) ^a^	QT Prolongation (2(7)) ^a^
Log-linear dose-response association model										
1 µg/L ^b^	1.000	1.000	1.000	1.000	1.000	1.000	1.000	1.000	1.000	1.000
3 µg/L	1.213 (1.070, 1.374)	1.079 (1.023, 1.139)	1.061 (0.891, 1.262)	1.176 (1.083, 1.276)	1.178 (1.108, 1.252)	1.051 (0.992, 1.114)	1.370 (1.175, 1.598)	1.104 (1.020, 1.195)	1.187 (0.848, 1.662)	1.363 (0.770, 2.414)
5 µg/L	1.327 (1.105, 1.593)	1.118 (1.034, 1.210)	1.090 (0.844, 1.407)	1.268 (1.125, 1.429)	1.272 (1.163, 1.391)	1.076 (0.989, 1.172)	1.587 (1.267, 1.987)	1.156 (1.030, 1.298)	1.286 (0.785, 2.105)	1.574 (0.682, 3.636)
10 µg/L	1.498 (1.153, 1.948)	1.174 (1.049, 1.313)	1.131 (0.784, 1.630)	1.405 (1.183, 1.667)	1.411 (1.242, 1.603)	1.111 (0.984, 1.254)	1.936 (1.403, 2.671)	1.231 (1.043, 1.452)	1.433 (0.707, 2.901)	1.914 (0.578, 6.339)
20 µg/L	1.693 (1.203, 2.380)	1.232 (1.064, 1.426)	1.173 (0.729, 1.889)	1.556 (1.245, 1.944)	1.566 (1.325, 1.848)	1.146 (0.979, 1.343)	2.362 (1.553, 3.590)	1.310 (1.057, 1.625)	1.597 (0.637, 3.998)	2.327 (0.490,11.052)
50 µg/L	1.988 (1.274, 3.103)	1.313 (1.085, 1.589)	1.233 (0.662, 2.295)	1.781 (1.331, 2.383)	1.796 (1.445, 2.230)	1.195 (0.973, 1.469)	3.071 (1.777, 5.308)	1.423 (1.074, 1.885)	1.842 (0.555, 6.109)	3.012 (0.394, 23.045)
coefficient	0.175	0.070	0.054	0.148	0.150	0.046	0.287	0.090	0.156	0.282
*p*-value for trend ^c^	0.003	0.005	0.510	< 0.001	< 0.001	0.090	< 0.001	0.014	0.320	0.290
I^2 d^	79.7%	77.9%	89.0%	6.6%	17.4%	0.0%	17.5%	62.3%	80.4%	91.5%
Cochran’s Q-statistic	29.54	31.70	54.78	3.21	1.21	2.88	2.43	18.56	5.10	11.7
P-heterogeneity ^e^	< 0.001	< 0.001	< 0.001	0.360	0.271	0.409	0.297	0.097	0.024	0.006
Non-linear dose-response association model (restricted cubic splines)										
1 µg/L^b^	1.000	1.000	1.000	1.000	1.000	1.000	1.000	1.000	1.000	1.000
3 µg/L	1.163 (1.060, 1.276)	0.999 (0.983, 1.014)	1.092 (0.862, 1.382)	0.985 (0.811, 1.197)	0.954 (0.647, 1.406)	1.026 (0.854, 1.232)	1.225 (0.783, 1.917)	1.012 (0.944, 1.085)	1.578 (0.707, 3.523)	1.070 (0.772, 1.483)
5 µg/L	1.250 (1.090, 1.433)	1.001 (0.980, 1.023)	1.136 (0.807, 1.596)	0.978 (0.735, 1.302)	0.933 (0.528, 1.648)	1.044 (0.815, 1.338)	1.347 (0.699, 2.594)	1.018 (0.920, 1.128)	1.951 (0.601, 6.326)	1.105 (0.685, 1.781)
10 µg/L	1.387 (1.135, 1.695)	1.015 (0.986, 1.043)	1.192 (0.746, 1.902)	0.986 (0.663, 1.468)	0.915 (0.410, 2.040)	1.081 (0.798, 1.464)	1.537 (0.612, 3.863)	1.027 (0.888, 1.187)	2.601 (0.483, 14.001)	1.155 (0.583, 2.288)
20 µg/L	1.557 (1.182, 2.052)	1.045 (1.012, 1.080)	1.241 (0.701, 2.195)	1.124 (0.720, 1.754)	0.963 (0.371, 2.499)	1.133 (0.816, 1.574)	1.800 (0.605, 5.353)	1.041 (0.868, 1.249)	3.449 (0.389, 30.605)	1.229 (0.504, 2.996)
50 µg/L	1.846 (1.231, 2.769)	1.125 (1.077, 1.176)	1.295 (0.659, 2.542)	1.795 (1.029, 3.131)	1.199 (0.439, 3.273)	1.220 (0.848, 1.753)	2.394 (0.852, 6.728)	1.082 (0.877, 1.334)	4.642 (0.298, 72.343)	1.433 (0.440, 4.667)
*p*-value for trend ^f^	0.006	< 0.001	0.750	0.047	0.078	0.390	< 0.001	0.200	0.150	0.270
I^2 d^	69.8%	35.3%	80.0%	41.0%	53.7%	0.0%	0.0%	46.3%	73.1%	72.5%
Cochran’s Q-statistic	39.75	21.65	60.02	10.16	4.32	5.65	2.58	26.07	7.43	7.27
P-heterogeneity ^e^	< 0.001	0.086	< 0.001	0.117	0.115	0.460	0.629	0.025	0.024	0.026

CVD: cardiovascular disease; CHD: coronary heart disease. a: Sum of studies included; the total number of relative risks in each model. b: treat 1 µg/L water arsenic concentration as the referent. c: *p*-value for linear trend from a Wald test of the coefficient for drinking water arsenic concentrations. d: Proportion of total variance due to between-study heterogeneity. e: *p*-value for heterogeneity is chi-square *p*-value of the Q-statistic. f: Non-linear trend *p*-value for the non-linear spline coefficient in a model with arsenic concentrations entered as a restricted cubic spline with knots at 10th, 50th and 90th percentiles of water arsenic concentration.

**Table 3:** Goodness-of-fit assessment.

Studies	Mortality Risk			Combined Fatal and non-Fatal Risk					CVD Markers	
	CHD	CVD	Stroke	CHD	CVD	Stroke	Carotid Atherosclerosis Disease	Hypertension	Pulse Blood Pressure	QT Prolongation
Log-linear dose-response association model										
Deviance ^a^	19.40	22.58	15.98	13.04	7.06	18.53	2.99	20.27	14.02	4.97
Degrees of freedom ^b^	17	22	17	9	4	11	5	21	6	4
*p*-value ^c^	0.306	0.426	0.526	0.161	0.133	0.070	0.702	0.504	0.029	0.291
R^2^	0.320	0.258	0.027	0.537	0.798	0.134	0.844	0.230	0.066	0.185
Adjusted R^2^	0.280	0.225	−0.031	0.486	0.748	0.056	0.813	0.193	−0.089	−0.019
AIC	0.17	−6.77	6.58	−0.56	1.26	−2.22	3.38	−4.36	4.55	5.58
Non-linear dose-response association model (restricted cubic splines)										
Deviance ^a^	17.28	22.81	15.39	5.83	3.94	17.61	1.71	12.94	9.16	3.44
Degrees of freedom ^b^	16	21	16	8	3	10	4	20	5	3
*p*-value ^c^	0.367	0.354	0.496	0.666	0.267	0.062	0.789	0.880	0.103	0.328
R^2^	0.373	0.620	0.035	0.512	0.564	0.097	0.892	0.199	0.292	0.435
Adjusted R^2^	0.297	0.584	−0.085	0.390	0.273	-0.084	0.838	0.118	0.008	0.058
AIC	29.95	5.89	23.86	12.34	10.37	16.07	13.75	23.55	12.16	11.43

CVD: cardiovascular disease; CHD: coronary heart disease. a: Measure of the total absolute deviation between reported and predicted log-relative risk taking into account the covariance structure of the residuals. b: Degrees of freedom from the deviance statistic. c: *p*-value from test for model specification. AIC: Akaike’s information criterion.

## Supplementary Materials

The following are available online at https://www.mdpi.com/1660-4601/17/23/8947/s1, Figure S1: Flow diagram of study selection procedure, Figure S2: Association of CVD endpoints with drinking water arsenic concentrations, Figure S3: Funnel Plots for the analysis of publication bias, Table S1: Epidemiological studies of arsenic (As) exposure and cardiovascular disease (CVD) included in the systematic review, Table S2: Egger’s regression test of funnel plot asymmetry, Table S3: Pooled relative risks (95% confidence intervals) for different CVD types and clinical markers in relation to drinking water arsenic concentrations with the exclusion of studies which do provide drinking water As concentrations directly, Table S4: Pooled relative risks (95% confidence intervals) for different CVD types and CVD markers in relation to drinking water arsenic concentrations lower than 100 ppb.
