# Supplementary material for: Positive Association of Cardiovascular Disease (CVD) with Chronic Exposure to Drinking Water Arsenic (As) at Concentrations below the WHO Provisional Guideline Value: A Systematic Review and Meta-analysis
Source: Int J Environ Res Public Health. 2020 Apr 7;17(7):2536. doi: 10.3390/ijerph17072536 (PMC7178156; doi:10.3390/ijerph17072536)
Supplement: Supplementary file 1 [file ijerph-17-02536-s001.pdf]

Supplementary Material:

Positive association of cardiovascular disease (CVD) with chronic exposure to drinking water arsenic (As) at concentrations below the WHO provisional guideline value: A systematic review and meta-analysis

Lingqian Xu[1], Debapriya Mondal[2*], and David A. Polya[1]

[1] Department of Earth and Environmental Sciences and Williamson Research Centre for Molecular Environmental Science, University of Manchester, Manchester, M13 9PL, UK

[2] School of Science, Engineering & Environment, University of Salford, Salford, M5 4WT, UK

* Correspondence: d.mondal@salford.ac.uk Phone: +44 161 295 4137

Table S1. Epidemiological studies of arsenic (As) exposure and cardiovascular disease (CVD) included in the systematic review.

NO	Study (Year)	design	population	N (follow-up)	exposure assessment	exposure categories	outcome	outcome ascertainment	adjustment factors
1	Chen et al. [1] (2011)	prospective study	Bangladesh, 11746 men and women in 2000	followed up for an average of 6. 6 years	well water arsenic (As) and spot urine As	well As (μg/L)<br>mean (range) median<br>3.7 (0.1-12) 2<br>35.9 (12-62) 34<br>102.5 (62-148) 101<br>265.7 (148-864) 237<br>baseline urinary creatinine adjusted As (μg/g of creatinine)<br>mean (range) median<br>68 (6-105) 69<br>150 (106-199) 150<br>264 (199-351) 262<br>641 (351-1100) 538	deaths from cardiovascular disease	defined as deaths from disease of circulatory system (ICD-10 (international classification of diseases, 10th revision) codes I00-I99)	sex and baseline age, BMI, smoking status, educational attainment, and changes in As concentration
2	Chen et al. [2] (2013)	prospective study	Bangladesh, recruited 20033 residents 18-75 years of age (original cohort) in 2000 at baseline. HEALS was expanded to include an additional 8,287 participants (expansion cohort) in 2007–2008	during 2005-2010, 5.9 years on average since baseline and followed with personal visits at 2-year intervals	As in drinking water and urine at baseline recruitment, and in follow-up urine samples collected every 2 years	well-water As (μg/L)<br>mean(range)<br>2.8 (0.1-9)<br>30.0 (9.5-57)<br>95.1 (58-144)<br>254.5 (145-790)<br>urinary As (μg/g creatinine)<br>mean(range)<br>66.1 (7-101)<br>140.8 (102-187)<br>249.7 (188-327)<br>606.3 (328-4306)	QTc prolongation	QT interval was measured from the beginning of the QRS complex to the end of the T wave, and was corrected for heart rate using the Bazzet formula.	sex and age, BMI, smoking status, and educational attainment, changes in urinary As between visits.
3	Chen et al. [3] (2007)	cross-sectional	10,910 participants in the Health Effects of As Longitudinal Study in Bangladesh (October 2000-May 2002)		time-weighted well As concentration (TWA) (μg/L)	range mean<br>0.1-8 2.8<br>8-40 23.2<br>40-91 63.9<br>91-176 128.1<br>176-864 283.1	general hypertension	general hypertension (SBP≥140 mmHg, and/or DBP≥90 mmHg), systolic hypertension (SBP≥140 mmHg), diastolic hypertension (DBP≥90 mmHg), and high pulse pressure (SBP-DBP≥55 mmHg)	age, gender, body mass index, cigarette smoking status, education length, and daily water consumption
4	Tsinovoi et al. [4] (2018)	case–cohort study	This sub-cohort (n = 2666) was selected from the entire	The average follow-up was 6.7 years	water As (μg/L)	median range<br>3.29 2.72-3.72<br>5.26 4.75-5.88<br>8.07 8.26-9.18	incident ischemic stroke	Cases of incident stroke were obtained every 6 months via	age at baseline, sex, race, age×race, and stroke region, body mass index, education,

			cohort of REGARDS participants			13.88<br>34.06	11.99-16.72<br>26.11-54.81			telephone and verified using medical record review.	smoking status, alcohol consumption, and physical activity, quintiles of urine cadmium and serum mercury
5	Sohel et al. [5] (2009)	perspective analysis	Matlab, Bangladesh; A total of 115,903 persons aged 15 or more years on 1 January 1991 were available for analysis.	followed them until 31 December 2000; 9,015 deaths; 22,488 lost to follow-up -	Study used average household exposure of As from drinking water (μg/L) as a proxy for individual exposure.	range<br>< 10<br>10-49<br>50-149<br>150-299<br>> 300	mean<br>1.4<br>31.0<br>97.0<br>208.6<br>402.5	median<br>0.7<br>31.8<br>95<br>201<br>371	cardiovascular disease	Cases were defined as persons within the cohort who had died of non-accidental causes during the period.	age, sex, asset score
6	D'Ippoliti et al. [6] (2015)	perspective study	165,609 residents of 17 municipalities	residents on January 1st 1990 and those who were subsequently born or immigrated to the municipality up to December 31th 2010	average individual As exposure at the first residence and time-dependent cumulative As dose indicator	range<br>< 10<br>10-20<br>> 20	As (μg/L)<br>mean<br>6.5<br>13.7<br>34.5<br><br>CAI (μg)<br>≤ 204.9<br>204.9-804.0<br>> 804.0	median<br>7.4<br>12.9<br>29.7	circulatory system diseases (390–459)	ischemic heart disease (410-414), myocardial infarction (410), coronary atherosclerosis (414), cerebrovascular diseases (430-438), stroke (430, 431, 434, 436), peripheral Arterial (440-448)	sex, age, calendar period, socioeconomic level, occupation in the ceramic industry, smoking sales and radon exposure
7	Medrano et al. [7] (2010)	ecological study	1721 municipalities located in 49 out of 52 Spanish provinces, covering 24.8 million people	NA	Tap drinking water As concentrations at the municipal level during 1998-2002 (μg/L) were obtained from the National Information System of Consume Water Control.	range<br>< 1<br>1-10<br>> 10	mean<br>0.7<br>3.9<br>23.3		cardiovascular mortality (CVD (ICD: I00 I99), CHD (ICD: I20 I25), and cerebrovascular diseases (ICD: I60 I69))	Cardiovascular mortality was analysed for the period 1999-2003. The observed number of deaths at the municipal level was obtained from the National Institute for Statistics.	sex, age, per capita municipal income, and hospital beds per population, smoking, hypertension, high serum cholesterol, diabetes, overweight/obesity, and low physical activity, fish, wine, olive oil, bottled water, and total energy and water characteristics at municipal level.
8	Moon et al. [8] (2013)	prospective study	3575 American Indian men and women aged 45 to 74 years living in Arizona, Oklahoma,	baseline visit between 1989 and 1991. Participants were invited to	sum of inorganic and methylated As species in urine at baseline (μg/g creatinine)	range (median)<br>< 5.8 (4.2)<br>5.8-9.7 (7.5)<br>9.8-15.7 (12.4)<br>15.7 (21.8)	mean<br>4.1<br>7.6<br>12.5<br>26.3		cardiovascular disease (incidence and mortality)	identified by annual contact, by review of hospitalization and death records, and	systolic blood pressure and hypertension medication use, AIC level, sex, age, education, smoking

			and North and South Dakota.	subsequent clinical visits in 1993-1995 and 1998-1999 and were actively followed through 2008,3575, 15 years						during 2 clinic visits conducted between 1993 and 1995 and between 1998 and 1999	status, body mass index, cholesterol level, hypertension, diabetes, and estimated glomerular filtration rate and albuminuria
9	Islam et al. [9] (2012)	cross-sectional study	rural Bangladesh, The study was conducted between January and July 2009	NA	As concentration in drinking water (µg/L)	range<br>10-22<br>23-32<br>33-261<br>≥ 262			hypertension prevalence and pulse pressure	Hypertension was defined as systolic blood pressure ≥ 140 mmHg (systolic hypertension) and diastolic blood pressure ≥ 90 mmHg (diastolic hypertension) and those with known hypertension and on antihypertensive medication. Pulse pressure was considered to be increased when the difference was ≥ 55 mmHg.	age, sex, education, marital status, religion, monthly income and BMI
10	James et al. [10] (2015)	case-cohort study	This study included 555 participants with no known coronary heart disease (CHD) events or diagnosis of DM before the baseline visit in Alamosa and Conejos counties of south central Colorado.	555 participants with 96 CHD events diagnosed between 1984 and 1998	time-weighted average inorganic As exposure (µg/L)	range<br>1-20<br>20-30<br>30-45<br>45-88	mean<br>7.31<br>25.1<br>36.6<br>50.2	median<br>5.71<br>25.3<br>35.1<br>50.5	identified CHD events	A CHD event was defined [ICD-9 codes 410-414]. Potential CHD events were identified through self-report and death certificate searches. The medical records were reviewed by a three-member committee of medical physician.	age, sex, BMI, physical activity, smoking status, alcohol consumption, serum lipid levels, and micronutrient intake
11	Li et al. [11] (2013)	cross-sectional study	604 of eligible subjects were confirmed, and	NA	cumulative arsenic exposure (CAE) in mg/L-year in the tube	urinary As species<br>iAs (µg/g Cr)<br>< 7.31<br>7.31 to 33.68	DMA (µg/g Cr)<br>< 66.70<br>66.70 to 181.85<br>> 181.85		hypertension	Hypertension was defined in this study as a systolic blood pressure ≥	gender, age, cigarette smoking, alcohol consumption and BMI.

			interviewed door to door.		wells and urinary As and its species	> 33.68<br>MMA (µg/g Cr)<br>< 11.28<br>11.28 to 37.89<br>> 37.89<br>tAs (µg/g Cr)<br>< 93.77<br>93.77 to 250.61<br>> 250.61<br>CAE (mg/L-year)<br>< 0.10<br>0.10 to 0.35<br>> 0.35		140 mm Hg, a diastolic blood pressure ≥ 90 mm Hg, or a history of hypertension under regular treatment with antihypertensive agents.	
12	Wade et al. [12] (2015)	hospital based case control study	A total of 298 cases and 275 controls were enrolled in the Bayingnormen (Ba Men) region of Inner Mongolia, China from a large hospital.	NA	toenail and drinking water As	water As (µg/L)<br>range / mean / median<br>< 10 / 3.02 / 1.91<br>10-39 / 20.87 / 16.03<br>40 and over / 78.75 / 58.57<br>nail As (µg/g)<br>range / mean / median<br>0.11-0.28 / 0.23 / 0.24<br>0.29-1.37 / 0.65 / 0.6<br>1.38-34.21 / 3.25 / 2.17	CHD incidence	acute myocardial infarction (mi), cardiomyopathy and chest pain chest pain suggestive of angina	age, sex, diet, body mass index (BMI); occupation; education; smoking; and family history of hypertension, diabetes or heart disease
13	Mumford et al. [13] (2007)	cross-sectional study	313 residents of the Ba Men region	NA	water As concentration (µg/L)	< 21<br>100-350<br>430-690	QT prolongation	QT interval defines the period of ventricular repolarization	age, sex, BMI, and age/BMI interaction
14	Mendez et al. [14] (2016)	cross-sectional study	A total of 1,160 adults were recruited in household visits between 2008 and 2012.	NA	household drinking water As concentrations, and total urinary speciated As	household drinking water As concentrations (µg/L)<br>< 25.5<br>≥ 25.5 to < 47.9<br>≥ 47.9 to < 79.0<br>≥ 79.0<br>total urinary speciated As (µg/L)<br>< 27.5<br>≥ 27.1 to < 55.8<br>≥ 55.8 to <105.0<br>≥ 105.0	CM risk markers	Elevated fasting levels of each lipid were defined as plasma TG ≥ 150 mg/dL, TC ≥ 200 mg/dL, and LDL ≥ 130 mg/dL. Fasting HDL < 40 mg/dL in men and < 50 mg/dL in women were designated as low. Hypertension was defined by systolic blood pressure (SBP) > 140 mmHg, diastolic blood pressure (DBP) > 90 mmHg, or self-reported use of anti-hypertensive medication.	age, sex, education, smoking status, alcohol consumption, recent seafood intake, weight status, elevated waist circumference, and main water source
15	Wu et al. [15] (2006)	case-control study	163 patients with carotid	NA	As concentration in well water	As concentration in well water<br>≤ 50.00	risk of carotid atherosclerosis	Indications of carotid	age and gender, addition of current

			atherosclerosis and 163 controls were studied from the Lanyang Basin of Ilan County in north-eastern Taiwan		(μg/L) and cumulative As exposure (μg/L-year)	50.01-100.00<br>≥ 100.01<br>cumulative As exposure<br>≤ 1.70<br>1.71-4.20 43<br>≥ 4.21			atherosclerosis were evaluated mainly based on 2 indices: the maximal ECCA intimal–medial thickness (IMT) and the presence of ECCA plaque.	smoking, total cholesterol, hypertension, and plasma homocysteine level
16	Hall et al. [16] (2017)	population based cancer case-control study	northern Chile; hypertension cases (n=612), and hypertension-free controls (n=654)	NA	cumulative As exposure; peak exposure; highest 5-year average exposure	lifetime cumulative exposure ([μg/L]-years)<br>< 2188<br>2188-7025<br>> 7025<br>peak exposure prior to 1971 (μg/L)<br>< 60<br>60-859<br>> 859<br>highest 5-year average prior to 1971 (μg/L)<br>< 60<br>60-559<br>> 559<br>lifetime highest 5-year average (μg/L)<br>< 60<br>60-623<br>> 623		prevalence of hypertension	Those self-reporting either a physician diagnosis of hypertension or use of an anti-hypertensive medication were classified as hypertension cases.	age, BMI, sex, and smoking
17	Rahman et al. [17] (1999)	cross-sectional study	Bangladesh. A total of 1595 adults (903 men and 578 women) had a history of As exposure, whereas 114 (50 men and 64 women) were unexposed.	NA	time weighted average As; As concentration-year	time weighted average As exposure (mg/L)<br>0<br>< 0.5<br>0.5 to 1.0<br>> 1.0	As concentration-year, mg-y/L 0<br>< 1.0<br>1.0-5.0<br>5.0-10.0<br>> 10.0	cases of hypertension	Hypertension was defined as a systolic blood pressure > 140 mm Hg combined with a diastolic blood pressure > 90 mm Hg.	age, sex, and BMI
18	Wang et al. [18] (2011)	perspective study	3 villages — Homei, Fuhsin, and Hsinming in Putai Township located on the south western coast of Taiwan. The original cohort consisted of 490 non-hypertensive residents in 1993.	Subjects were invited for health check-ups in 1993, 1996, and 2002/03. By 2002/03, 382 (78%) of these subjects were successfully followed and 138 had been lost to follow-up	As level and its species of drinking water and urine as well as cumulative As level	cumulative As level (mg/L year)<br>< 5.6<br>5.6-15.6<br>> 15.6<br>As conc. in well water (μg/L)<br>< 538<br>538-700<br>> 700<br>As(V) (μg/g creatinine)<br>< 1.17<br>1.17-2.67<br>> 2.67		incidence of hypertension	Hypertension (systolic BP > 140mmHg, diastolic BP > 90, or on anti-hypertensive therapy) was used to define cases, utilizing hypertension and related data collected at 1989-90 (baseline) and	age, gender, BMI, and glucose (≥ 6.11 mmol/l) adjusted

									2002-03 (follow-up).	
19	Wade et al. [19] (2009)	retrospective study	Each family in Ba Men provided names and demographic characteristics of all family members residing in the household between January 1, 1997 and December 1, 2004.	NA	water As level (µg/L)	range<br>0-5<br>5.1-20<br>20.1-100<br>100.1-300<br>Over 300	mean<br>1.6<br>11.9<br>38.8<br>168.2<br>421.1	median<br>1<br>11<br>26<br>156<br>387	heart disease mortality and stroke mortality	A team of medical experts evaluated the evidence available and coded each underlying cause of death according to the ICD-10 system.	age, sex education, smoking, alcohol use, farm work
20	Wang et al. [20] (2005)	a follow-up study in Taiwan	This study enrolled 10,133 and 16,718 residents aged 40 and older from arsenic-exposed and unexposed areas respectively.	NA	water As (µg/L)	range<br><10<br>10-49<br>50-499 ≥500	mean<br>5<br>29.5<br>274.5<br>724.5	median<br>5<br>29.5<br>274.5<br>724.5	CVD mortality	Deaths from ischemic heart disease and stroke were ascertained up to December 31, 2004 through linkage with national death certification profiles.	age, gender
21	Rahman et al. [21] (2014)	prospective study	Matlab, Bangladesh; recruited 61,074 adults	Participants were followed from January 01, 2003 until December 31, 2010 (~ 7 years).	TWA individual drinking water (µg/L)	range<br>< 10<br>10-49<br>>50		median<br>1.7<br>21.1<br>101.2	mortality risks of stroke	Stroke deaths: Verbal autopsy (ICD-10: I61–69)	age, sex, education attainment and SES
22	Chen et al. [22] (2013)	case–cohort study	369 incident fatal and nonfatal cases of CVD, including 211 cases of heart disease and 148 cases of stroke, and a sub-cohort of 1,109 subjects randomly selected from the original cohort study	The cohort continues to be actively followed every 2 years.	baseline well-water As (µg/L)	range<br>0.1-25<br>25.1-107<br>108-864	mean<br>7.2<br>59.9<br>222.8	median<br>5.1<br>57<br>198.5	CVD, heart disease and stroke cases risk (incidence)	incident fatal and nonfatal cases of CVD (ICD-10 codes I00-I99), including fatal and nonfatal stroke (codes I60-I69) and fatal and nonfatal cases of heart disease, which occurred after baseline and before 18 March 2009	sex, baseline age, BMI, smoking status, educational attainment, hypertension, diabetes status, and change in urinary As between visit
23	Hsieh et al. [23] (2008)	case-control study	A random sample of 479 subjects inclusive of 235 cases and 244 controls were selected.	NA	As concentration in well water and cumulative As exposure	As concentration in well water (µg/L)<br>≤ 10<br>10.1-50.0<br>≥ 50.1<br>cumulative As exposure (mg/L-year)<br>≤ 0.2			carotid atherosclerosis	Indications of carotid atherosclerosis were evaluated mainly based on three indices: the	age, gender, cigarette smoking, diabetes mellitus, cholesterol and triglyceride

						0.3-1<br>≥ 1.1				intima media thickness (IMT), the plaque score and the maximal level of stenosis of ECCA.	
24	Hsieh et al. [24] (2011)	community-based case-control study	A random sample of 863 subjects who had been genotyped for PNP, As3MT, GSTO1, and GSTO2 were selected with 384 subjects being defined as cases and the remaining 479 subjects categorized as reference group.	NA	As concentration in well water of the household (μg/L)	< 10<br>10.1-50.0<br>> 50.0			carotid atherosclerosis	Three indices including intima media thickness (IMT), the plaque score, and the maximal level of stenosis of the ECCA were determined as indications of carotid atherosclerosis.	age, gender, cigarette smoking, alcohol consumption, hypertension, cholesterol, fasting glucose, and body-mass index
25	Jones et al. [25] (2011)	cohort study	4167 participants for this study.	A total of 15,955 adults 20 years of age or older participated in NHANES between 2003 and 2008, leaving 4167 participants for this study.	Total urinary As (μg/L) and its species (μg/L)	total As<br>< 4.2<br>4.2 to 8.3<br>> 8.3 to 17.1<br>> 17.1<br>total As minus arsenobetaine<br>< 3.1<br>3.1 to 5.8<br>> 5.8 to 10.8<br>> 10.8<br>dimethylarsinate<br>< 2.0<br>2.0 to 3.6<br>> 3.6 to 6.0<br>> 6.0			hypertension and blood pressure	Hypertension was defined as a mean systolic blood pressure ≥ 140 mmHg, a mean diastolic blood pressure ≥ 90 mmHg, a self-reported physician diagnosis, or use of antihypertensive medication.	sex, age, race and ethnicity, and urine creatinine level, education, body mass index, serum cotinine level, and antihypertensive medication use and arsenobetaine
26	Chen et al. [26] (1996)	prospective	SW Taiwan 40–70 y 52% men	2556 (~5 y)	average concentration of As in drinking water (μg/L)	range<br>< 10<br>10-500<br>≥ 510	mean<br>5<br>255<br>755	median<br>5<br>255<br>755	developing lethal ISHD	national death registry (ICD-9: 410-414)	age, sex, blackfoot disease, status, cigarette smoking, body mass index, serum levels of cholesterol and triglycerides, and disease status for hypertension and diabetes
27	Farzan et al. [27] (2015)	prospective analysis of population-based non-	New Hampshire, USA Median 61 y 56% men	3939 (14 y)	toenail (μg/g)	range<br>0.01-0.07<br>0.07-0.11<br>0.11-3.26	mean<br>0.05<br>0.09<br>0.23	median<br>0.05<br>0.09<br>0.23	CVD, CHD and stroke mortality	national death index (ICD-10: I00-99, I20-25, I60-69)	age, sex, education, smoking, cancer status

		melanoma skin cancer case-control study								
28	Ersboll et al. [28] (2018)	prospective study	Copenhagen and Aarhus , a study population of 53,941 individuals	53,941 (12.8 years)	20-year time weighted average (TWA) As concentration in drinking water (μg/L)	range<br>0.049-0.573<br>0.573-0.760<br>0.760-1.933<br>1.933-25.34	median<br>0.435<br>0.584<br>1.174<br>2.109	incidence rate of all strokes	Stroke was defined based on International Classification of Disease (ICD) ICD-8 codes: 430, 431, 433, 434, 436.01, or 436.90 until 1994 and ICD-10 codes: I60, I61, I63 or I64 from 1994.	age, sex, body mass index, waist circumference, smoking status, smoking duration, smoking intensity, alcohol status, intake of alcohol, physical activity, fruit intake, vegetable intake, length of school attendance, and calendar year

Table S2. Egger's regression test of funnel plot asymmetry.

	Mortality risk			Combined fatal and non-fatal risk					CVD markers	
	CHD	CVD	Stroke	CHD	CVD	Stroke	Carotid atherosclerosis disease	Hypertension	Pulse blood pressure	QT prolongation
z	5.088	2.161	1.569	1.589	NA	1.030	1.551	0.722	NA	NA
p-value	< 0.001	0.030	0.117	0.112	NA	0.303	0.121	0.470	NA	NA

CVD: cardiovascular disease; CHD: coronary heart disease.

Notes: Calculated using the 'metafor' package in R.

NA: Egger's test only conducted for models with at least three studies.

Table S3. Pooled relative risks (95% confidence intervals) for different CVD types and clinical markers in relation to drinking water arsenic concentrations with the exclusion of studies which do not provide drinking water As concentrations directly.

Drinking water arsenic concentration	Mortality risk			Combined fatal and non-fatal risk		
	CHD (5(18)) [a]	CVD (7(24)) [a]	Stroke (5(18)) [a]	CHD (3(10)) [a]	Stroke (3(12)) [a]	Hypertension (7(26)) [a]
Log-linear dose-response association model						
1 μg/L[b]	1.000	1.000	1.000	1.000	1.000	1.000
3 μg/L	1.175<br>(1.026, 1.345)	1.060<br>(1.015, 1.107)	1.016<br>(0.860, 1.199)	1.209<br>(1.060, 1.378)	1.042<br>(0.982, 1.105)	1.103<br>(1.009, 1.207)
5 μg/L	1.267<br>(1.039, 1.544)	1.090<br>(1.023, 1.161)	1.023<br>(0.801, 1.305)	1.321<br>(1.090, 1.600)	1.062<br>(0.973, 1.158)	1.155<br>(1.013, 1.317)
10 μg/L	1.403<br>(1.056, 1.863)	1.131<br>(1.033, 1.239)	1.033<br>(0.729, 1.464)	1.489<br>(1.131, 1.960)	1.090<br>(0.962, 1.234)	1.229<br>(1.019, 1.483)
20 μg/L	1.553<br>(1.074, 2.247)	1.174<br>(1.043, 1.321)	1.044<br>(0.663, 1.643)	1.679<br>(1.175, 2.400)	1.118<br>(0.951, 1.315)	1.308<br>(1.025, 1.669)
50 μg/L	1.777<br>(1.097, 2.878)	1.233<br>(1.057, 1.439)	1.058<br>(0.584, 1.912)	1.968<br>(1.234, 3.138)	1.157<br>(0.937, 1.429)	1.420<br>(1.033, 1.952)
p-value for trend [c]	0.019	0.008	0.850	0.004	0.180	0.031
I^2 [d]	79.8%	78.0%	91.9%	35.2%	0.0%	66.4%
Cochran's Q-statistic	19.83	22.74	49.51	3.08	0.969	17.84
P-heterogeneity [e]	< 0.001	< 0.001	< 0.001	0.213	0.615	0.007
AIC	1.61	-7.91	3.02	2.40	-1.84	-1.94
Non-linear dose-response association model (restricted cubic splines)						
1 μg/L[b]	1.000	1.000	1.000	1.000	1.000	1.000
3 μg/L	1.152<br>(1.033, 1.285)	0.999<br>(0.983, 1.014)	1.032<br>(0.826, 1.291)	1.041<br>(0.820, 1.322)	0.982<br>(0.770, 1.254)	1.004<br>(0.946, 1.066)
5 μg/L	1.231<br>(1.049, 1.446)	1.002<br>(0.980, 1.023)	1.047<br>(0.758, 1.446)	1.061<br>(0.747, 1.505)	0.984<br>(0.714, 1.357)	1.006<br>(0.922, 1.098)
10 μg/L	1.349<br>(1.071, 1.699)	1.015<br>(0.987, 1.044)	1.066<br>(0.682, 1.664)	1.112<br>(0.687, 1.800)	1.002<br>(0.688, 1.458)	1.008<br>(0.890, 1.142)
20 μg/L	1.481<br>(1.089, 2.012)	1.044<br>(1.011, 1.079)	1.081<br>(0.624, 1.872)	1.297<br>(0.753, 2.233)	1.032<br>(0.697, 1.529)	1.011<br>(0.860, 1.189)
50 μg/L	1.680<br>(1.090, 2.587)	1.118<br>(1.070, 1.168)	1.095<br>(0.569, 2.106)	2.147<br>(1.009, 4.565)	1.087<br>(0.725, 1.630)	1.027<br>(0.839, 1.257)
p-value for trend [c]	0.039	< 0.001	0.960	0.120	0.650	0.240
I^2 [d]	71.6%	24.8%	85.3%	46.6%	0.0%	42.6%
Cochran's Q-statistic	28.19	13.30	54.4	7.49	3.60	20.91
P-heterogeneity [e]	< 0.001	0.207	< 0.001	0.112	0.461	0.052
AIC	18.49	-5.24	8.52	12.57	15.16	17.24

CVD: cardiovascular disease; CHD: coronary heart disease.

a: Sum of studies included; the total number of relative risks in each model.

b: treat 1 μg/L water arsenic concentration as the referent.

c: P-value for linear trend from a Wald test of the coefficient for water arsenic concentrations.

d: Proportion of total variance due to between-study heterogeneity.

e: P-value for heterogeneity is chi-square p-value of the Q-statistic.
f: Non-linear trend p-value for the non-linear spline coefficient in a model with water arsenic concentrations entered as a restricted cubic spline with knots at 10th, 50th and 90th percentiles.

Table S4. Pooled relative risks (95% confidence intervals) for different CVD types and CVD markers in relation to drinking water arsenic concentrations lower than 100 ppb.

Drinking water arsenic concentration	Mortality risk			Combined fatal and non-fatal risk			
	CHD (4(13))[a]	CVD (4(13))[a]	Stroke (4(13))[a]	CHD (3(11))[a]	Stroke (3(13))[a]	Carotid atherosclerosis disease (2(6))[a]	Hypertension (2(8))[a]
Log-linear dose-response association model							
1 μg/L[b]	1.000	1.000	1.000	1.000	1.000	1.000	1.000
3 μg/L	1.248 (1.010, 1.544)	1.151 (0.971, 1.366)	1.224 (0.933, 1.605)	1.214 (1.078, 1.367)	1.044 (0.981, 1.112)	1.313 (1.115, 1.546)	1.153 (1.001, 1.327)
5 μg/L	1.384 (1.014, 1.889)	1.230 (0.958, 1.579)	1.345 (0.904, 2.000)	1.329 (1.117, 1.582)	1.066 (0.972, 1.169)	1.490 (1.174, 1.892)	1.232 (1.002, 1.514)
10 μg/L	1.593 (1.021, 2.485)	1.345 (0.940, 1.922)	1.528 (0.866, 2.696)	1.502 (1.171, 1.927)	1.095 (0.960, 1.250)	1.770 (1.257, 2.491)	1.347 (1.003, 1.810)
20 μg/L	1.832 (1.027, 3.269)	1.470 (0.923, 2.341)	1.736 (0.829, 3.634)	1.699 (1.228, 2.348)	1.126 (0.948, 1.336)	2.101 (1.347, 3.278)	1.474 (1.004, 2.164)
50 μg/L	2.206 (1.036, 4.697)	1.654 (0.901, 3.036)	2.055 (0.783, 5.394)	1.997 (1.308, 3.0496)	1.167 (0.933, 1.460)	2.637 (1.475, 4.713)	1.659 (1.005, 2.740)
p-value for trend[c]	0.040	0.100	0.140	0.001	0.180	0.001	0.048
I^2 [d]	89.3%	80.2%	79.6%	25.1%	23.2%	0.0%	0.0%
Cochran's Q-statistic	18.69	15.11	14.74	2.67	2.60	0.07	0.14
P-heterogeneity[e]	< 0.001	0.001	0.002	0.263	0.272	0.791	0.709
AIC	3.69	2.81	6.54	2.19	0.84	2.16	1.95
Non-linear dose-response association model (restricted cubic splines)							
1 μg/L[b]	1.000	1.000	1.000	1.000	1.000	1.000	1.000
3 μg/L	1.343 (1.029, 1.751)	1.029 (0.996, 1.064)	1.880 (0.805, 4.389)	0.958 (0.762, 1.204)	1.041 (0.897, 1.208)	1.464 (0.903, 2.376)	1.137 (0.930, 1.390)
5 μg/L	1.528 (1.039, 2.247)	1.055 (0.996, 1.118)	2.433 (0.738, 8.011)	0.939 (0.672, 1.313)	1.071 (0.875, 1.312)	1.749 (0.861, 3.552)	1.207 (0.899, 1.620)
10 μg/L	1.774 (1.037, 3.034)	1.130 (0.989, 1.290)	3.063 (0.688, 13.624)	0.937 (0.588, 1.493)	1.131 (0.852, 1.500)	2.214 (0.819, 5.987)	1.311 (0.870, 1.974)
20 μg/L	1.999 (1.016, 3.932)	1.260 (0.966, 1.643)	3.363 (0.677, 16.699)	1.133 (0.682, 1.884)	1.207 (0.809, 1.801)	2.723 (0.846, 8.757)	1.434 (0.891, 2.309)
50 μg/L	2.310 (0.978, 5.454)	1.483 (0.928, 2.368)	3.575 (0.677, 18.860)	2.109 (1.176, 3.780)	1.322 (0.729, 2.397)	3.277 (1.117, 9.620)	1.638 (0.975, 2.752)
p-value for trend[f]	0.047	0.180	0.290	0.005	0.650	0.004	0.140
I^2 [d]	81.0%	67.0%	65.2%	0.1%	0.0%	0.0%	0.0%
Cochran's Q-statistic	31.57	18.81	17.21	4.00	3.12	0.41	0.37
P-heterogeneity[e]	< 0.001	0.005	0.008	0.405	0.537	0.81	0.827
AIC	25.97	17.01	24.59	11.86	13.85	8.77	12.10

CVD: cardiovascular disease; CHD: coronary heart disease.

a: Sum of studies included; the total number of relative risks in each model.

b: treat 1 μg/L water arsenic concentration as the referent.

c: P-value for linear trend from a Wald test of the coefficient for water arsenic concentrations.

d: Proportion of total variance due to between-study heterogeneity.

e: P-value for heterogeneity is chi-square p-value of the Q-statistic.

f: Non-linear trend p-value for the non-linear spline coefficient in a model with water arsenic concentrations entered as a restricted cubic spline with knots at 10th, 50th and 90th percentiles.

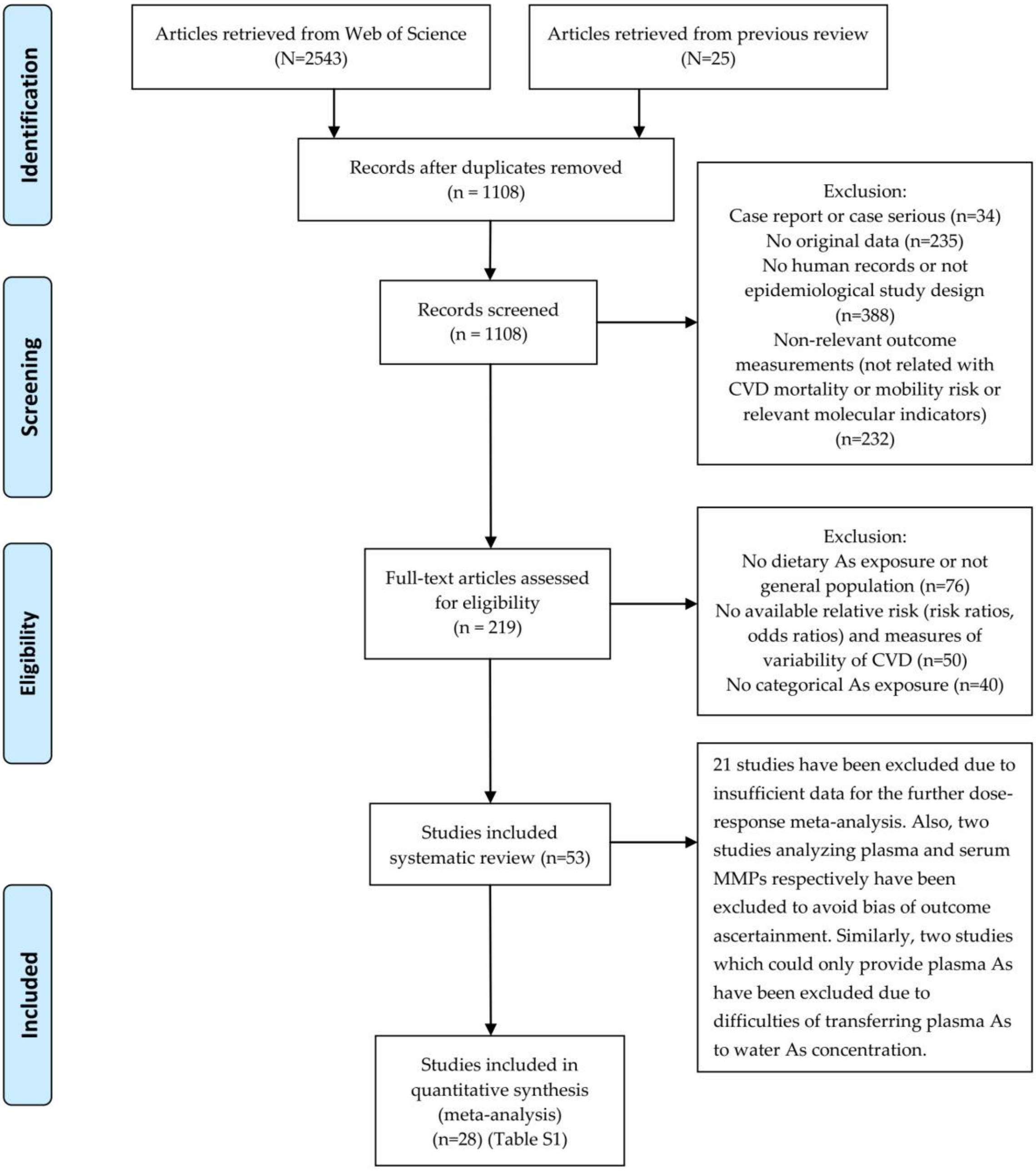

Figure S1. Flow diagram of study selection procedure.

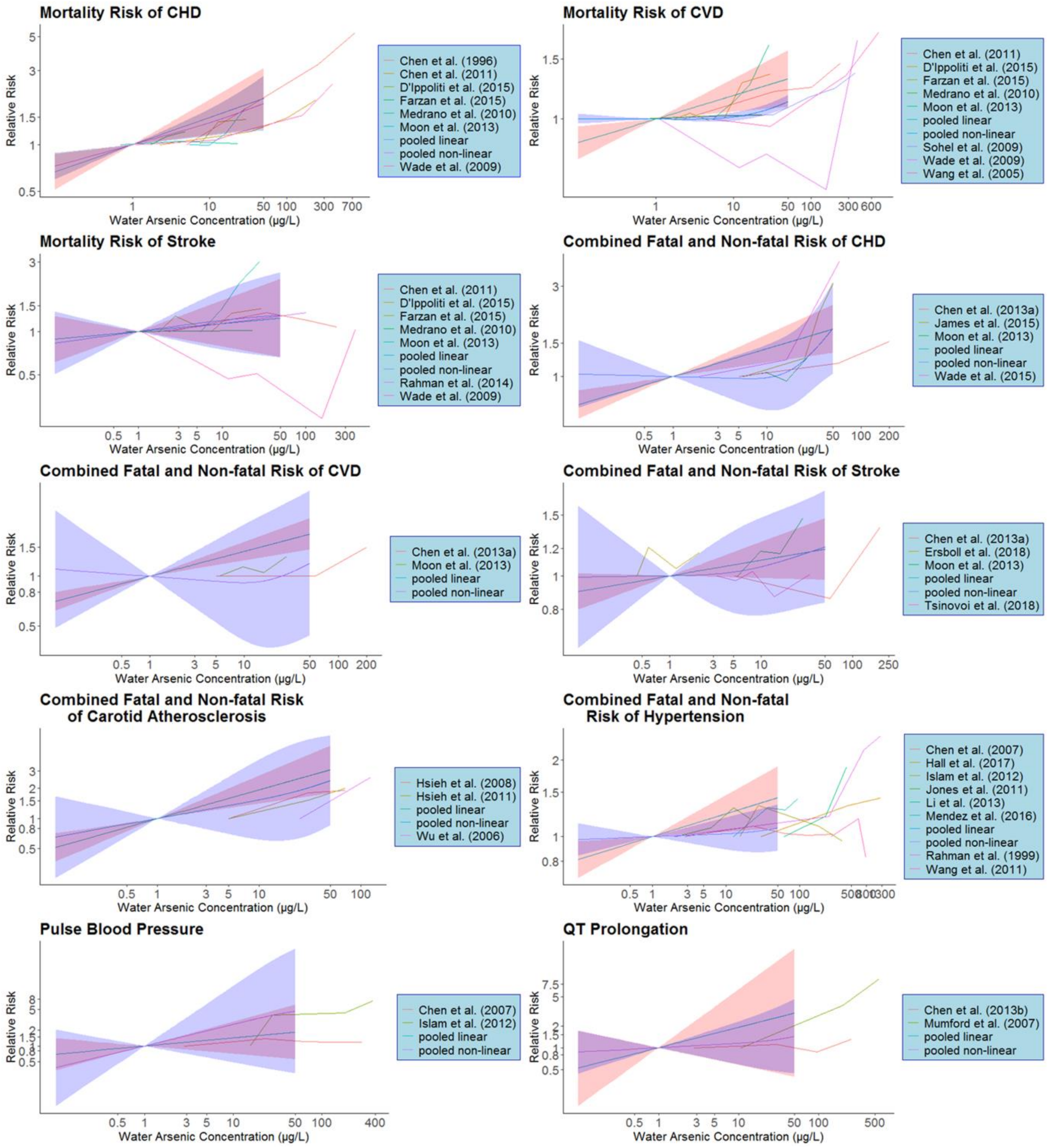

Figure S2. Association of CVD endpoints with drinking water arsenic concentrations. Dose-response relationships for individual studies were overprinted by the pooled dose-response relationship for each CVD endpoint to visually test the model goodness-of-fit. Shaded area represents the 95 % confidence intervals of log-linear model (red) and non-linear model (blue) (CVD: cardiovascular disease; CHD: coronary heart disease).

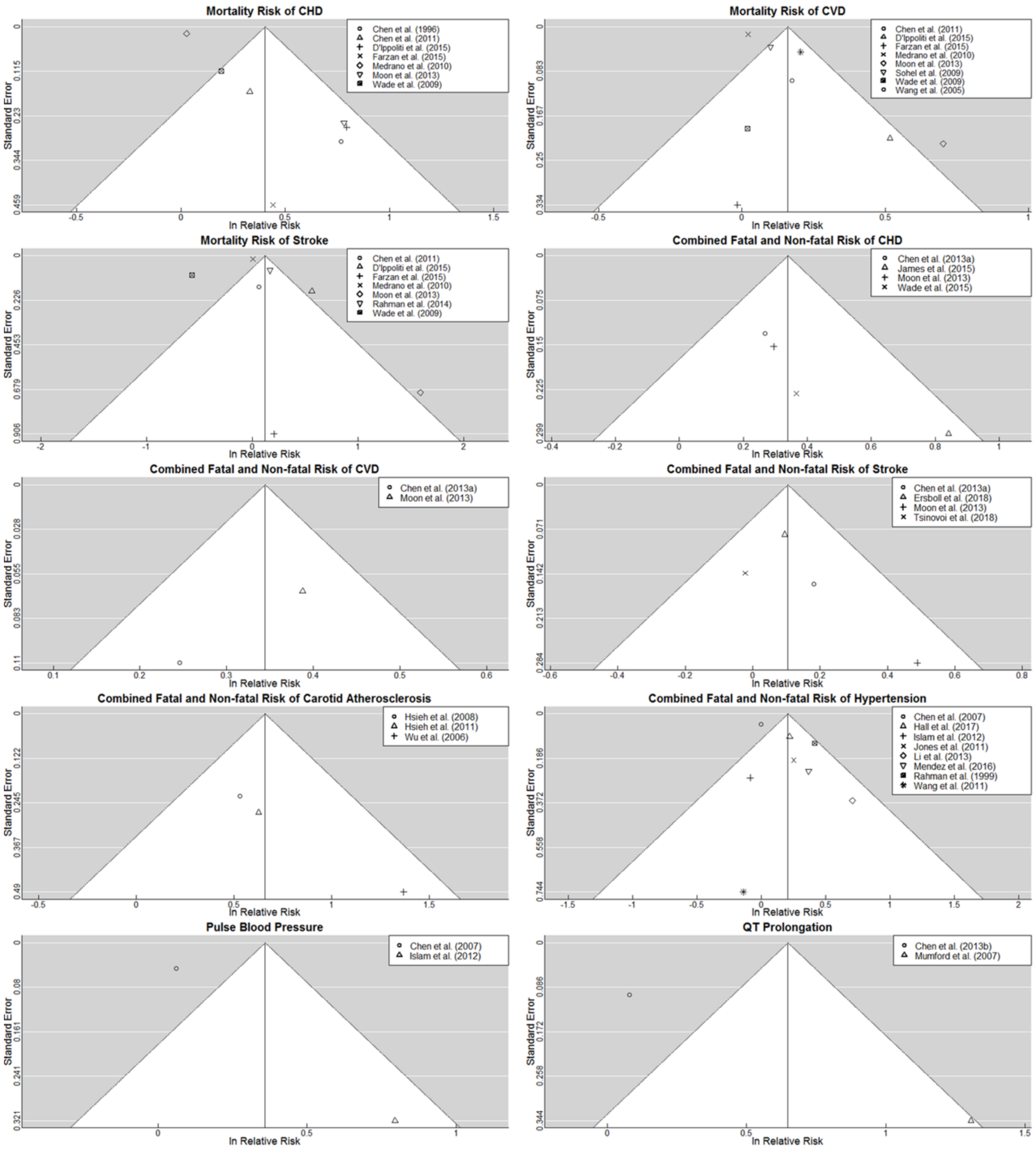

Figure S3. Funnel Plots for the analysis of publication bias. Funnel plots of the pooled linear dose-response models for each CVD endpoint. In this study, funnel plots were created using the metafor package in R. Each funnel plot was cantered at the overall model estimate, with the effect estimated from each study (log- relative risk) plotted against the accordingly standard error. Shaded area represents the region in which 95% of the study points might be expected to lie without the presence of both heterogeneity and publication bias (CVD: cardiovascular disease; CHD: coronary heart disease).

References

1. Chen, Y.; Graziano, J.H.; Parvez, F.; Liu, M.; Slavkovich, V.; Kalra, T.; Argos, M.; Islam, T.; Ahmed, A.; Rakibuzzaman, M. Arsenic exposure from drinking water and mortality from cardiovascular disease in Bangladesh: prospective cohort study. *BMJ: British Medical Journal (Overseas & Retired Doctors Edition)* **2011**, *342*, d2431, doi:10.1136/bmj.d2431.
2. Chen, Y.; Wu, F.; Parvez, F.; Ahmed, A.; Eunus, M.; McClintock, T.R.; Patwary, T.I.; Islam, T.; Ghosal, A.K.; Islam, S. Arsenic exposure from drinking water and QT-interval prolongation: results from the Health Effects of Arsenic Longitudinal Study. *Environmental health perspectives* **2013**, *121*, 427-432.
3. Chen, Y.; Factor-Litvak, P.; Howe, G.R.; Graziano, J.H.; Brandt-Rauf, P.; Parvez, F.; van Geen, A.; Ahsan, H. Arsenic exposure from drinking water, dietary intakes of B vitamins and folate, and risk of high blood pressure in Bangladesh: A population-based, cross-sectional study. *American Journal of Epidemiology* **2007**, *165*, 541-552, doi:10.1093/aje/kwk037.
4. Tsinovoi, C.L.; Xun, P.C.; McClure, L.A.; Carioni, V.M.O.; Brockman, J.D.; Cai, J.W.; Guallar, E.; Cushman, M.; Unverzagt, F.W.; Howard, V.J., et al. Arsenic Exposure in Relation to Ischemic Stroke The Reasons for Geographic and Racial Differences in Stroke Study. *Stroke* **2018**, *49*, 19-26, doi:10.1161/strokeaha.117.018891.
5. Sohel, N.; Persson, L.A.; Rahman, M.; Streatfield, P.K.; Yunus, M.; Ekstrom, E.C.; Vahter, M. Arsenic in Drinking Water and Adult Mortality A Population-based Cohort Study in Rural Bangladesh. *Epidemiology* **2009**, *20*, 824-830, doi:10.1097/EDE.0b013e3181bb56ec.
6. D'Ippoliti, D.; Santelli, E.; De Sario, M.; Scortichini, M.; Davoli, M.; Michelozzi, P. Arsenic in Drinking Water and Mortality for Cancer and Chronic Diseases in Central Italy, 1990-2010. *Plos One* **2015**, *10*, e0138182, doi:10.1371/journal.pone.0138182.
7. Medrano, M.J.; Boix, R.; Pastor-Barriuso, R.; Palau, M.; Damian, J.; Ramis, R.; del Barrio, J.L.; Navas-Acien, A. Arsenic in public water supplies and cardiovascular mortality in Spain. *Environmental Research* **2010**, *110*, 448-454, doi:10.1016/j.envres.2009.10.002.
8. Moon, K.A.; Guallar, E.; Umans, J.G.; Devereux, R.B.; Best, L.G.; Francesconi, K.A.; Goessler, W.; Pollak, J.; Silbergeld, E.K.; Howard, B.V., et al. Association Between Exposure to Low to Moderate Arsenic Levels and Incident Cardiovascular Disease. *Annals of Internal Medicine* **2013**, *159*, 649-659, doi:10.7326/0003-4819-159-10-201311190-00719.
9. Islam, M.R.; Khan, I.; Attia, J.; Hassan, S.M.N.; McEvoy, M.; D'Este, C.; Azim, S.; Akhter, A.; Akter, S.; Shahidullah, S.M., et al. Association between Hypertension and Chronic Arsenic Exposure in Drinking Water: A Cross-Sectional Study in Bangladesh. *International Journal of Environmental Research and Public Health* **2012**, *9*, 4522-4536, doi:10.3390/ijerph9124522.
10. James, K.A.; Byers, T.; Hokanson, J.E.; Meliker, J.R.; Zerbe, G.O.; Marshall, J.A. Association between Lifetime Exposure to Inorganic Arsenic in Drinking Water and Coronary Heart Disease in Colorado Residents. *Environmental Health Perspectives* **2015**, *123*, 128-134, doi:10.1289/ehp.1307839.
11. Li, X.; Li, B.; Xi, S.H.; Zheng, Q.M.; Wang, D.; Sun, G.F. Association of urinary monomethylated arsenic concentration and risk of hypertension: a cross-sectional study from arsenic contaminated areas in northwestern China. *Environmental Health* **2013**, *12*, 37-46, doi:10.1186/1476-069x-12-37.
12. Wade, T.J.; Xia, Y.J.; Mumford, J.; Wu, K.G.; Le, X.C.; Sams, E.; Sanders, W.E. Cardiovascular disease and arsenic exposure in Inner Mongolia, China: a case control study. *Environmental Health* **2015**, *14*, 35-44, doi:10.1186/s12940-015-0022-y.
13. Mumford, J.L.; Wu, K.G.; Xia, Y.J.; Kwok, R.; Wang, Z.H.; Foster, J.; Sanders, W.E. Chronic arsenic exposure and cardiac repolarization abnormalities with QT interval prolongation in a population-based study. *Environmental Health Perspectives* **2007**, *115*, 690-694, doi:10.1289/ehp.9686.
14. Mendez, M.A.; Gonzálezhorta, C.; Sánchezramírez, B.; Ballinascasarrubias, L.; Cerón, R.H.; Morales, D.V.; Terrazas, F.A.B.; Ishida, M.C.; Gutiérreztorres, D.S.; Saunders, R.J. Chronic Exposure to Arsenic and Markers of Cardiometabolic Risk: A Cross-Sectional Study in Chihuahua, Mexico. *Environmental Health Perspectives* **2016**, *124*, 104-111, doi:10.1289/ehp.1408742.
15. Wu, M.M.; Chiou, H.Y.; Hsueh, Y.M.; Hong, C.T.; Su, C.L.; Chang, S.F.; Huang, W.L.; Wang, H.T.; Wang, Y.H.; Hsieh, Y.C., et al. Effect of plasma homocysteine level and urinary monomethylarsonic acid on the

risk of arsenic-associated carotid atherosclerosis. *Toxicology and Applied Pharmacology* **2006**, *216*, 168-175, doi:10.1016/j.taap.2006.05.005.

16. Hall, E.M.; Acevedo, J.; Lopez, F.G.; Cortes, S.; Ferreccio, C.; Smith, A.H.; Steinmaus, C.M. Hypertension among adults exposed to drinking water arsenic in Northern Chile. *Environmental Research* **2017**, *153*, 99-105, doi:10.1016/j.envres.2016.11.016.
17. Rahman, M.; Tondel, M.; Ahmad, S.A.; Chowdhury, I.A.; Faruquee, M.H.; Axelson, O. Hypertension and arsenic exposure in Bangladesh. *Hypertension* **1999**, *33*, 74-78, doi:10.1161/01.hyp.33.1.74.
18. Wang, S.L.; Li, W.F.; Chen, C.J.; Huang, Y.L.; Chen, J.W.; Chang, K.H.; Tsai, L.Y.; Chou, K.M. Hypertension incidence after tap-water implementation: A 13-year follow-up study in the arseniasis-endemic area of southwestern Taiwan. *Science of the Total Environment* **2011**, *409*, 4528-4535, doi:10.1016/j.scitotenv.2011.07.058.
19. Wade, T.J.; Xia, Y.; Wu, K.; Li, Y.; Ning, Z.; Le, X.C.; Lu, X.; Feng, Y.; He, X.; Mumford, J.L. Increased mortality associated with well-water arsenic exposure in Inner Mongolia, China. *Int J Environ Res Public Health* **2009**, *6*, 1107-1123, doi:10.3390/ijerph6031107.
20. Wang, C.H.; Chen, C.L.; Hsu, L.I.; Chiou, H.Y.; Hsueh, Y.M.; Chen, S.Y.; Wu, M.M.; Hsiao, C.K. *Chronic Arsenic Exposure Increases Mortality from Ischemic Heart Disease and Stroke: A Follow-up Study on 26,851 Residents in Taiwan*; National Taiwan University: Taipei, Taiwan, 2005.
21. Rahman, M.; Sohel, N.; Yunus, M.; Chowdhury, M.E.; Hore, S.K.; Zaman, K.; Bhuiya, A.; Streatfield, P.K. A prospective cohort study of stroke mortality and arsenic in drinking water in Bangladeshi adults. *BMC Public Health* **2014**, *14*, 1-8, doi:10.1186/1471-2458-14-174.
22. Chen, Y.; Wu, F.; Liu, M.L.; Parvez, F.; Slavkovich, V.; Eunus, M.; Ahmed, A.; Argos, M.; Islam, T.; Rakibuz-Zaman, M., et al. A Prospective Study of Arsenic Exposure, Arsenic Methylation Capacity, and Risk of Cardiovascular Disease in Bangladesh. *Environmental Health Perspectives* **2013**, *121*, 832-838, doi:10.1289/ehp.1205797.
23. Hsieh, Y.C.; Hsieh, F.I.; Lien, L.M.; Chou, Y.L.; Chiou, H.Y.; Chen, C.J. Risk of carotid atherosclerosis associated with genetic polymorphisms of apoliploprotein E and inflammatory genes among arsenic exposed residents in Taiwan. *Toxicology and Applied Pharmacology* **2008**, *227*, 1-7, doi:10.1016/j.taap.2007.10.013.
24. Hsieh, Y.C.; Lien, L.M.; Chung, W.T.; Hsieh, F.I.; Hsieh, P.F.; Wu, M.M.; Tseng, H.P.; Chiou, H.Y.; Chen, C.J. Significantly increased risk of carotid atherosclerosis with arsenic exposure and polymorphisms in arsenic metabolism genes. *Environmental Research* **2011**, *111*, 804-810, doi:10.1016/j.envres.2011.05.003.
25. Jones, M.R.; Tellezplaza, M.; Sharrett, A.R.; Guallar, E.; Navasacien, A. Urine Arsenic and Hypertension in U.S. Adults: the 2003–2008 NHANES. *Epidemiology* **2011**, *22*, 153-161, doi:10.1097/EDE.0b013e318207fdf2.
26. Chen, C.J.; Chiou, H.Y.; Chiang, M.H.; Lin, L.J.; Tai, T.Y. Dose-response relationship between ischemic heart disease mortality and long-term arsenic exposure. *Arteriosclerosis Thrombosis and Vascular Biology* **1996**, *16*, 504-510, doi:10.1161/01.atv.16.4.504.
27. Farzan, S.F.; Chen, Y.; Rees, J.R.; Zens, M.S.; Karagas, M.R. Risk of death from cardiovascular disease associated with low-level arsenic exposure among long-term smokers in a US population-based study. *Toxicology and Applied Pharmacology* **2015**, *287*, 93-97, doi:10.1016/j.taap.2015.05.013.
28. Ersboll, A.K.; Monrad, M.; Sorensen, M.; Baastrup, R.; Hansen, B.; Bach, F.W.; Tjonneland, A.; Overvad, K.; Raaschou-Nielsen, O. Low-level exposure to arsenic in drinking water and incidence rate of stroke: A cohort study in Denmark. *Environment International* **2018**, *120*, 72-80, doi:10.1016/j.envint.2018.07.040.
